# Structural determination of oleanane-28,13β-olide and taraxerane-28,14β-olide fluoro­lactonization products from the reaction of oleanolic acid with Selectfluor^TM^

**DOI:** 10.1107/S2056989024006480

**Published:** 2024-07-15

**Authors:** Megan A. Eadsforth, Linghan Kong, George Whitehead, Iñigo J. Vitórica-Yrezábal, Raymond T. O’Keefe, Richard A. Bryce, Roger C. Whitehead

**Affiliations:** a Department of Chemistry, University of Manchester, Manchester, United Kingdom; b Division of Pharmacy and Optometry, University of Manchester, Manchester, United Kingdom; c Department of Inorganic Chemistry, Faculty of Science, University of Granada, Granada, Spain; d Division of Evolution, Infection and Genomics, University of Manchester, Manchester, United Kingdom; University of Massachusetts Dartmouth, USA

**Keywords:** crystal structure, oleanolic acid, fluoro­lactone, selectfluor, taraxerane, oleanane, triterpenoids, lactone

## Abstract

The X-ray crystal structure data of 12-α-fluoro-3β-hy­droxy­olean-28,13β-olide methanol hemisolvate, 2C_30_H_47_FO_3_·CH_3_OH, (**1**), and 12-α-fluoro-3β-hy­droxy­taraxer-28,14β-olide methanol hemisolvate, 2C_30_H_47_FO_3_·CH_3_OH, (**2**), are described. The fluoro­lactonization of oleanolic acid using Selectfluor^TM^ yielded a mixture of the six-membered δ-lactone (**1**) and the unusual seven-membered γ-lactone (**2**) following a 1,2-shift of methyl C-27 from C-14 to C-13.

## Abstract 2

X-ray analysis and structure determination of fluoro­lactonization products from the reaction of oleanolic acid with Selectfluor^TM^ are reported.

## 1. Chemical context

Oleanolic acid (OA) and ursolic acid (UA) are penta­cyclic triterpenoids that are found widely in food and plants of the Oleaceae family, such as the olive plant. Similar to many other natural products, these triterpenoids have been found to exhibit a range of pharmacological activities (Sánchez-Quesada *et al.*, 2013 ▸), such as anti­oxidant, anti-inflammatory (Adjei *et al.*, 2021 ▸), anti-diabetic (Qian *et al.*, 2010 ▸; Tang *et al.*, 2014 ▸), and anti-cancer properties (Borella *et al.*, 2019 ▸; Baer-Dubowska *et al.*, 2021 ▸). Previous reports have demonstrated that semi-synthetic derivatives of UA and OA-fluoro­lactones have improved biological activities compared to the parent mol­ecules, demonstrating both anti-apoptotic (Leal *et al.*, 2012 ▸) and anti-diabetic properties (Zhong *et al.*, 2019 ▸). Leal and co-workers report that the reaction of UA with Selectfluor^TM^ at 353 K, in a mixture of nitro­methane and dioxane, results in insertion of fluorine at C-12 with formation of the 28,13β-γ-lactone. The authors report that fluoro­lactonization of UA gives a mixture of α and β-isomers (C-F), with the β-isomer formed as the major product, as characterized by ^1^H NMR spectroscopy (Leal *et al.*, 2012 ▸). Zhong and co-workers (Zhong *et al.*, 2019 ▸) also report fluoro­lactonization of OA, under these same conditions (Leal *et al.*, 2012 ▸), to give fluorination at C-12 and formation of the 28,13β-β-lactone. However, the authors (Zhong *et al.*, 2019 ▸) do not comment on the stereochemistry at the C—F bond (C-12).

**Figure 1:**
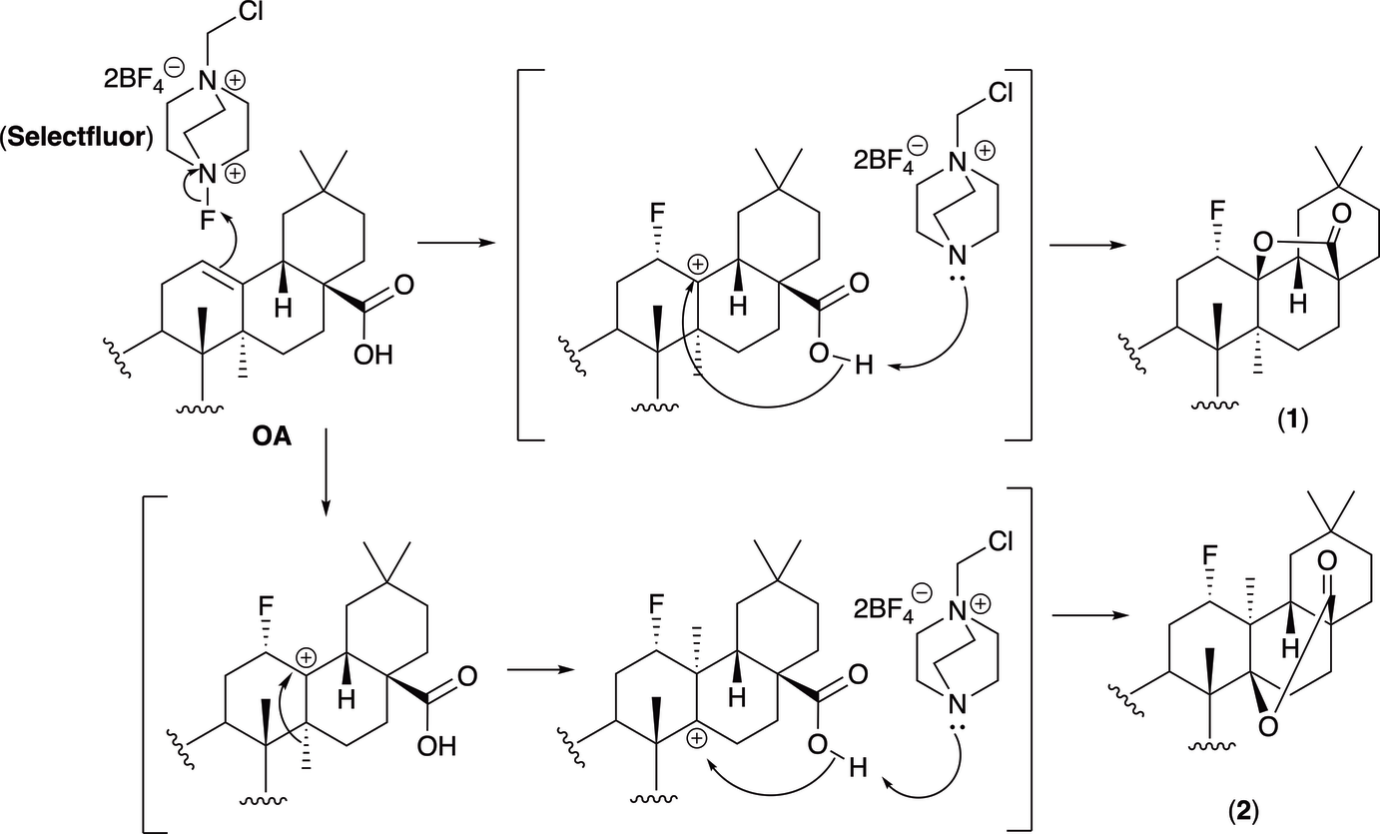
Proposed mechanism for the formation of oleanolic fluoro­lactone products (**1**) and (**2**).

Given the previous reports indicating enhanced pharmacological properties in UA and OA-fluoro­lactone derivatives (Leal *et al.*, 2012 ▸; Zhong *et al.*, 2019 ▸), our research pursuits include the additional functionalization of OA-fluoro­lactones. This exploration aims to unveil alternative biological activities within this class of compounds (Eadsforth, 2022 ▸). We therefore adopted the same conditions as previously described for UA (Leal *et al.*, 2012 ▸) and OA (Zhong *et al.*, 2019 ▸) for the fluoro­lactonization of OA (see scheme[Chem scheme1]). Inter­estingly, we found that under these conditions a mixture of fluoro­lactone products was formed, including the 28,14β-δ-lactone, which has not previously been characterized (Fig. 1 ▸). Herein, we report on the synthesis and crystal structures of the products of the fluoro­lactonization reaction of OA with Selectfluor^TM^, an electrophilic fluorinating reagent, which leads to rearrangement to a taraxerane core as the major product. Taraxeranes are another class of biologically active penta­cyclic triterpenoid steroids that have been isolated from plants. Taraxeranes are structural isomers of oleanane triterpenoids that are derived bio-synthetically from the rearrangement of the oleanane skeleton following the C-27 methyl shift from C-14 to C-13 (Kuroda *et al.*, 2006 ▸; Hu *et al.*, 2012 ▸).

The classical lactonization reaction of oleanane-type triterpenoids, containing a C12=C13 double bond, has been reported to proceed under acidic conditions and involves a 28,13β-lactonization (Cheriti *et al.*, 1994 ▸). The reaction of oleanolic acid with bis­muth tri­fluoro­methane­sulfonate, Bi(OTf)_3_·*x*H_2_O, in DCM also results in 28,13β-lactonization, as confirmed by X-ray crystallography (Salvador *et al.*, 2009 ▸; Santos *et al.*, 2010 ▸). Our observation that fluoro­lactonization of OA with Selectfluor results in the formation of the 28,14β-δ-lactone has not yet been reported. However, our findings parallel previous reports in the literature which report formation of other oleanane-type 28,13β-δ-lactone derivatives under different conditions. For instance, using NMR analysis for characterization, the formation of 12-bromo-3β-hy­droxy­taraxeran-28,14β-olide as a minor product from the reaction of OA with bromine in CCl_4_ has been reported (Martinez *et al.*, 2015 ▸). The formation of 3-*O*-acetyl-taraxeran-28,14β-olide from the reaction of OA under oxidative conditions (formic acid/hydrogen peroxide at 373 K for several hours) has also been reported and characterized by NMR analysis (Heise *et al.*, 2021 ▸). X-ray analysis has also identified 3β-acet­oxy-12α-chloro-14β-isooleanan-28,14β-olide as an unexpected by-product in a POCl_3_-catalysed Beckmann rearrangement of 3β-acet­oxy-12-hy­droxy­imino­olean-28-olic acid methyl ester (Froelich *et al.*, 2011 ▸).
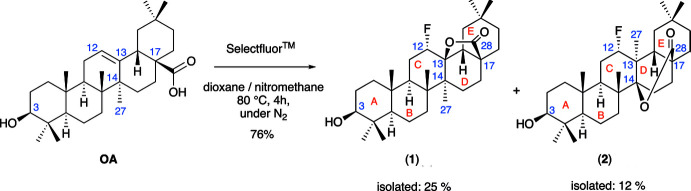


While the precise mechanism underlying the formation of compound (**2**) following reaction of OA with Selectfluor^TM^ remains unclear, Martinez and co-workers have previously elucidated a possible mechanism for the formation 12-bromo-3β-hy­droxy­taraxeran-28,14β-olide (Martinez *et al.*, 2015 ▸). Following bromination of oleanolic acid with Br_2_/CCl_4_, the authors report isolation of 12α-bromo-3β-hy­droxy­olean-28,13β-olide (80%), together with 12α-bromo-3β-hy­droxy­taraxer-14-en-28-oic acid (3%), and 12α-bromo-3β-hy­droxy­olean-28,13β-olide (13%), as minor products when the reaction was maintained for 12 h. The authors propose that formation of 12α-bromo-3β-hy­droxy­taraxer-14-en-28-oic acid could be explained due to traces of HBr in the reaction mixture leading to lactone ring-opening and formation of the C-28 carboxyl group. The subsequent migration of C-27 from C-14 to C-13, *via* the α-face, followed by elimination of H-15β would install a double bond between C-14 and C-15. Further addition of a proton to the double bond at C-14/C-15 would create a tertiary carbocation formed at C-14, which would be stabilized by the attack of the carboxyl group at C-28 to form 12-α-bromo-3β-hy­droxy­olean-28,13β-olide.

Since acid catalysis is unlikely under the electrophilic fluorinating conditions employed with Selectfluor^TM^ and given that we failed to isolate any inter­mediates containing a C-14=C-15 double bond, we propose that formation of (**2**) follows a different mechanism to that proposed for the bromo­lactonization of OA (Martinez *et al.*, 2015 ▸). We propose that following electrophilic addition of fluorine to the C12=C13 double bond, the tertiary carbocation formed at C-13 can either be stabilized by the intra­molecular nucleophilic attack of the C-28 carboxyl to form (**1**) or by the Wagner–Meerwein 1,2-shift of C-27 to C-13. This rearrangement would result in a tertiary carbocation at C-14, which can then be stabilized by the nucleophilic attack of the C-28 carboxyl group to form the 28,14β-δ-lactone (**2**) (Fig. 1 ▸).

## 2. Structural commentary

**Figure 2:**
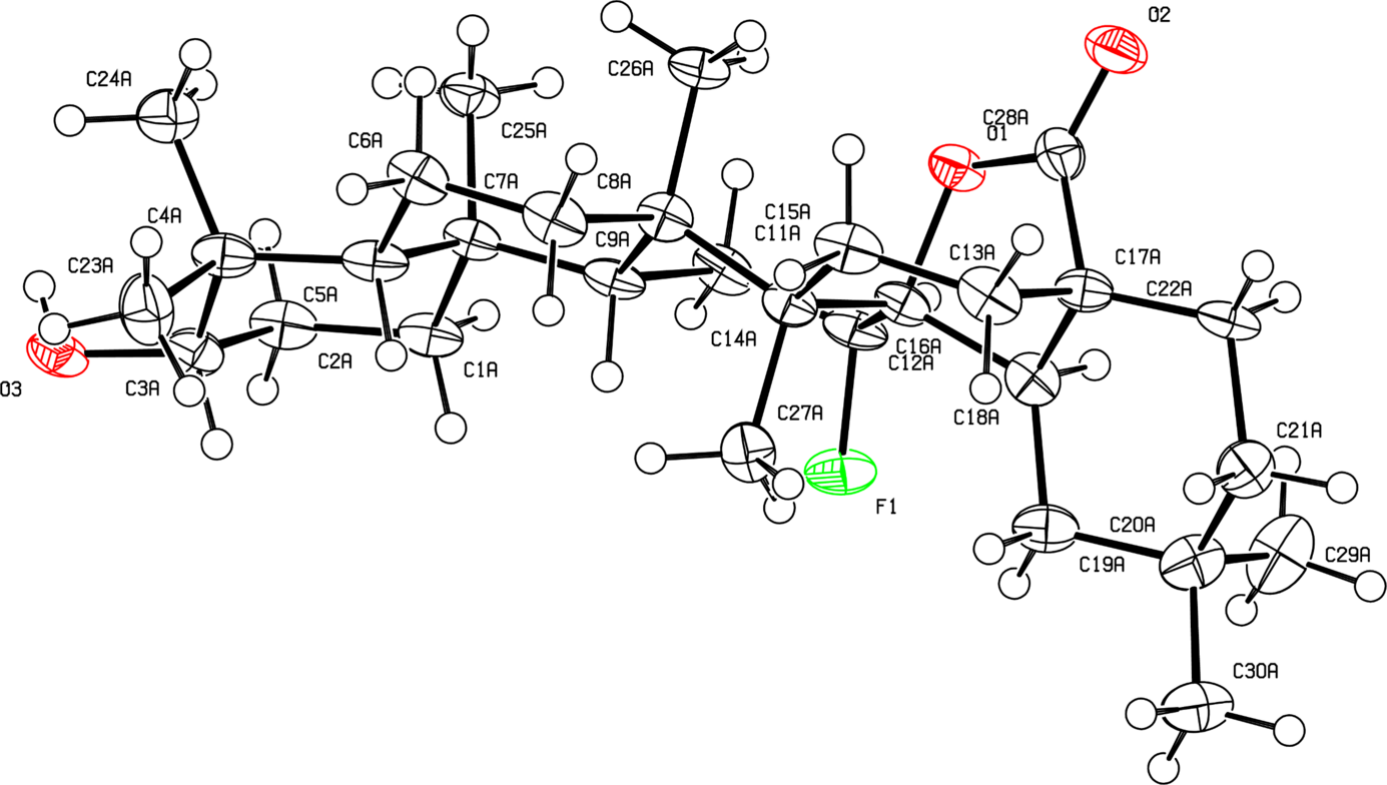
The mol­ecular structure of (**1**), showing the atomic labelling scheme. Non-H atoms are drawn as 50% probability displacement ellipsoids and H atoms are drawn as spheres of an arbitrary size. Oxygen atoms are coloured in red, fluorine atom coloured in green.

X-ray analysis of the OA-fluoro­lactonization products identified 3β-hy­droxy-12α-fluoroolean-28,13β-olide methanol hemisolvate (**1**) and 3β-hy­droxy-12α-fluorotaraxeran-28,14β-olide methanol hemisolvate (**2**) as the two main products. Both compounds (**1**) and (**2**) contain five fused six-membered rings. The X-ray structure of compound (**1**) revealed that it contains a γ-lactone ring with the C28=O2 carbonyl adjacent to C-17 and the bridging oxygen atom O-1 adjacent to C-13. The ether oxygen atom O-1 at C-13 and the methyl group at C-14 are axial with respect to rings *C* and *D*. The fluorine atom at C-12, belonging to ring *C*, is oriented equatorially and assumes an α-configuration. Rings *A*–*E* of the triterpenoid skeleton adopt chair conformations, and rings *D* and *E* are *cis*-fused as in oleanolic acid (Fig. 2 ▸). The values of the dihedral angles in (**1**) confirm the *trans* configuration of rings *A*/*B*, *B*/*C* and *C*/*D* [−177.9, 172.3, −170.8 (6)°] and the *cis* configuration of rings *D*/*E* [65.1°]. Each six-membered ring adopts a chair conformation with a different degree of distortion, as shown by the Cremer & Pople (1975 ▸) parameters: [ring *A*: *Q* = 0.552 Å, θ = 3.8° and φ = 312°; *B*: *Q* = 0.547 Å, θ = 166.2° and φ = 4.6°; *C*: *Q* = 0.550 Å, θ = 20.4° and φ = 132.3°; *D*: *Q* = 0.607 Å, θ = 161.2° and φ = 231.3°; *E*: *Q* = 0.570 Å, θ = 177.5° and φ = 247.5°]. The lactone ring adopts an envelope conformation (*q* = 0.487 and φ = 68.0°) as in agreement with the structure of 3-oxo-18a-olean-28,13β-olide (Santos *et al.*, 2010 ▸).

**Figure 3:**
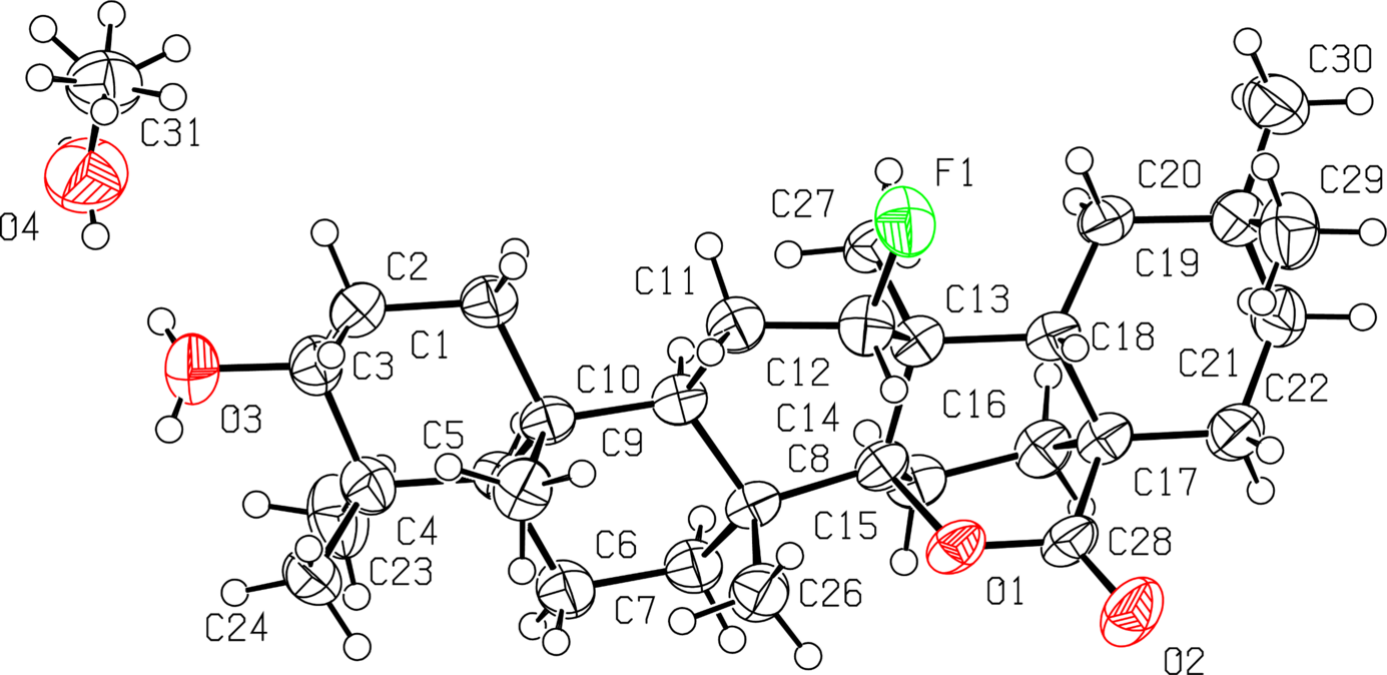
The mol­ecular structure of (**2**), showing the atomic labelling scheme. Non-H atoms are drawn as 50% probability displacement ellipsoids and H atoms are drawn as spheres of an arbitrary size. Methanol solvent mol­ecule hydrogen bonding to O-3. Oxygen atoms are coloured in red, fluorine atom coloured in green.

X-ray analysis confirmed that the unusual major product, compound (**2**), contains a δ-lactone ring; in (**2**), the C28=O2 carbonyl is adjacent to C-17 and the bridging oxygen atom O-1 adjacent to C-14 (Fig. 3 ▸). The ether oxygen displays a β-configuration, while the methyl group has an α-configuration. The structure shows that the methyl group C-27 has undergone a 1,2-shift from C-14 to C-13, retaining its original axial orientation from oleanolic acid. The fluorine atom at C-12, belonging to ring *C*, is oriented equatorially and assumes an α-configuration. Rings *A*, *B* and *E* of the triterpenoid skeleton adopt chair conformations [Cremer & Pople, 1975 ▸; puckering parameters: *Q* = 0.564 Å, θ = 176.8, φ = 241.1, *Q* = 0.586 Å, θ = 171.8, φ = 358.0, *Q* = 0.538, Å, θ = 171.6, φ = 20.7, respectively] whilst rings *C*, *D* and the δ-lactone group adopt twisted-boat conformations [puckering parameters: *Q* = 0.738 Å, θ = 93.9°, φ = 157.0°, *Q* = 0.852 Å, θ = 88.4°, φ = 165.6°, *Q* = 0.808 Å, θ = 91.7°, φ = 250.2°, respectively], in agreement with the 3β-acet­oxy-12α-chloro-14β-isooleanan-28,14β-olide structure (Froelich *et al.*, 2011 ▸). The values of the dihedral angles in compound (**2**) confirm the *trans* configuration of rings *A*/*B*, *B*/*C* and *C*/*D* [−179.3, −179.3, 164.8 (4)°] and the *cis* configuration of rings *D*/*E* [48.0°]. The hy­droxy group at C-3 in both structures (**1**) and (**2**) adopts a β-configuration.

## 3. Supra­molecular features

**Figure 4:**
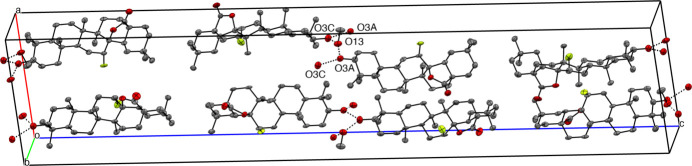
Expanded view of crystal packing of structure (**1**) to show hydrogen bonding (dotted lines) to solvent methanol. Oxygen atoms are coloured in red, fluorine atom coloured in green. The hydrogen atoms have been omitted for clarity.

**Figure 5:**
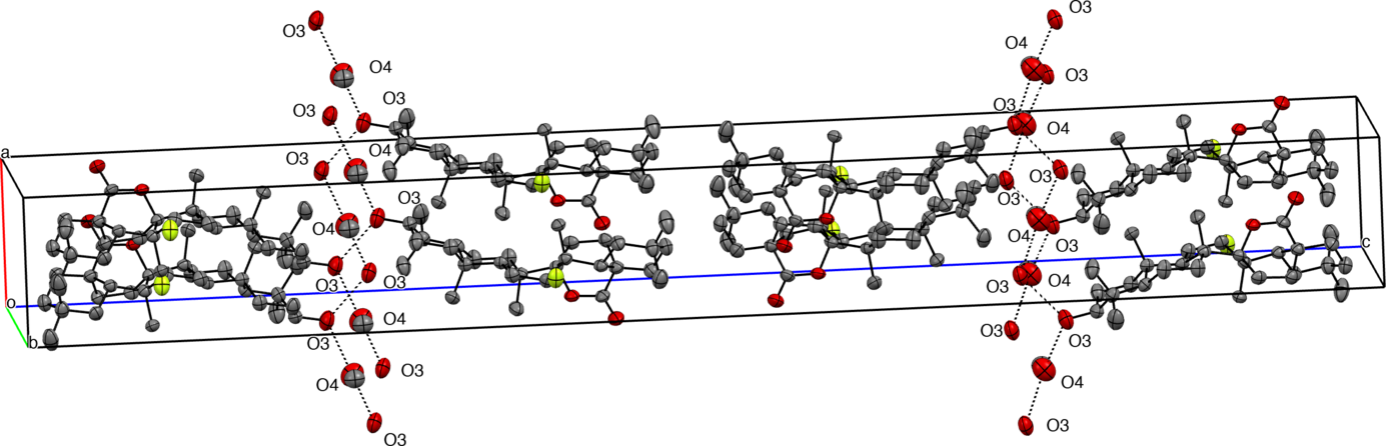
Expanded view of crystal packing of structure (**2**) to show hydrogen bonding (dotted lines) to solvent methanol. Oxygen atoms are coloured in red, fluorine atom coloured in green. The hydrogen atoms have been omitted for clarity.

In the crystal, two mol­ecules of compound (**1**) are connected by inter­molecular O—H⋯O hydrogen bonds between O-3 and a bridging mol­ecule of methanol (O3*A*⋯H13—O13⋯H12—O12) into infinite chains extending along [010] *b* axis (Fig. 4 ▸, Table 1 ▸). In the crystal structure of (**2**), the mol­ecules are connected by inter­molecular O—H⋯O hydrogen bonds between O-3 and a mol­ecule of methanol (O4—H4⋯O3) into infinite chains extending along [100] (Fig. 5 ▸, Table 2 ▸).

## 4. Synthesis and crystallization

Oleanolic acid (300 mg, 0.66 mmol) and Selectfluor^TM^{1-chloro­methyl-4-fluoro-1,4-diazo­niabi­cyclo­[2.2.2]octane bis­(tetra­fluoro­borate)} (701 mg, 1.98 mmol) were dissolved in a mixture of anhydrous dioxane (4 mL) and nitro­methane (6 mL), under an inert atmosphere, and stirred at 353 K for 4 h. The reaction mixture was then diluted with water (50 mL), extracted with ethyl acetate (3 × 50 mL) and the combined organic extract was washed with brine (3 × 20 mL). The organic phase was then de-emulsified by filtering through a sinter funnel containing a layer of NaCl. The organic phase was dried over anhydrous MgSO_4_, filtered and evaporated to dryness to afford an off-white solid. ^19^F NMR analysis of the crude mixture showed the ratio of products (**1**):(**2**) to be ∼1:1.25, indicating (**2**) to be the major isomer formed under these reaction conditions.

The crude product (239 mg, 0.50 mmol, 76%) was then dry-loaded on a silica gel column and purified with gradient elution (10–20%, EtOAc in hexa­ne), to afford products (**1**) (80 mg, 0.168 mmol, 25%) and (**2**) (38 mg, 0.080 mmol, 12%) as white crystalline solids. Due to poor separation by silica gel column chromatography, (**1**) was isolated in greater yield than (**2**). Recrystallization of (**1**) by methanol evaporation afforded colourless needle-like crystals suitable for X-ray diffraction analysis. Further purification of (**2**) by recrystallization in aceto­nitrile, followed by evaporation from methanol provided colourless crystals suitable for X-ray diffraction analysis.

**12α-Fluoro-3β-hy­droxy­olean-28,13β-olide methanol hemisolvate (1):**

White solid; m.p. 563 K; [α]D^22^ +43.3 (*c* 1.00 in DCM). ν_max_ cm^−1^ ∼3490 *w* (O—H), 2924m (C—H), 1773 (C=O,γ-lactone); δ_H_ (400 MHz, CDCl_3_) 0.68–0.74 (1 H, *m*, C5H), 0.75 (3 H, *s*, C24H_3_), 0.85 (3 H, *s*, C25H_3_), 0.88 (3 H, *s*, C30H_3_), 0.97 (7 H, *s*, C1*A*H, C23H_3_ and C29H_3_), 1.09 (3 H, *s*, C26H_3_), 1.21 (3 H, *s*, C27H_3_),1.12–2.14 [20 H, *m*, C(1*B*, 9H) and C(2, 6, 7, 1, 15, 16, 19, 21, 22)H_2_], 3.20 (1 H, *dd*, *J* 11.3, 4.7, C3H), 4.54 (1 H, *dt*, ^1^*J*_C–F_ 46.6, 2.8, C12H_F_). δ_C_ (101 MHz, CDCl_3_) 15.5 (C24), 16.1 (C25), 17.8 (C6), 18.1 (*d*, ^4^*J*_C–F_ 8.1, C27), 18.4 (C26), 21.2 (C16), 23.9 (C30), 25.8 (*d*, ^2^*J*_C–F_ 21.5, C11), 27.3 (C2), 27.5 (C15), 27.6 (C22), 28.1 (C23), 31.6 (C20), 33.3 (C29), 33.6 (C(7), 34.2 (C21), 36.5 (C10), 38.6 (C1 & C19 overlapping peaks), 39.0 (C4), 41.8 (*d*, ^3^*J*_C–F_ 1.3, C14), 42.0 (C8), 44.4 (C17), 44.8 (C9), 51.0 (C18), 55.1 (C5), 78.8 (C3), 88.1 (*d*, ^2^*J*_C–F_ 25.9, (C13), 96.8 (*d*, ^1^*J*_C–F_ 171.6, (C12), 179.4 (C28). δ_F_ (376 MHz, CDCl_3_) −180.0 (*td*, ^1^*J*_C–F_ 47.1, 20.4). *m*/*z* HR–MS[ESI^+^] calculated for C_30_H_48_FO_3_ [(*M*+H)^+^] 475.3587, found 475.3582.

**12-α-Fluoro-3β-hy­droxy­taraxer-28,14β-olide methanol hemisolvate (2):**

White needles; m.p. 526 K (charring); [α]D^22^ +25.7 (*c* 1.00 in DCM). ν_max_ cm^−1^ 3490 *w* (O—-H), 2935 *m* (C—-H), 1737 *s* (C=O, δ-lactone). δ_H_ (400 MHz, CDCl_3_) 0.76 (1 H, *d*, *J* 2.9, C5H), 0.79 (3 H, *s*, C24H_3_), 0.88 (3 H, *s*, C30H_3_), 0.93 (3 H, *s*, C25H_3_), 0.97–1.05 (1 H, *m*, C1*A*H), 0.96 (6 H, *s*, C23H_3_ and C29H_3_), 1.13 (3 H, *s*, C26H_3_), 1.22 (3 H, *d*, ^4^*J*_C–F_ 2.6, C27H_3_), 1.14–1.32 (3 H, *m*, C16*A*H and C21H_2_), 1.33–1.46 [4 H, *m*, C22H_2_ and (7*A*, 19*A*)H], 1.50–1.77 [8 H, *m*, C2, 6H_2_ and C(1*B*, 7*B*, 11*A*, 15*A*H)], 1.82–1.89 (2 H, *m*, C9, 18H), 1.90–2.05 (2 H, *m*, C11*B*, 15*B*H), 2.09 (1 H, *td*, *J* 14.2, 4.4, C19*B*H), 2.15–2.26 (1 H, *m*, C16*B*H), 3.14–3.24 (1 H, *m*, C3H), 4.65 (1 H, *ddd*, ^1^*J*_C–F_ 50.8, 6.7, 4.1, C12H_F_). δ_C_ (126 MHz, CDCl_3_) 15.3 (C24), 16.6 (*d*, ^3^*J*_C–F_ 10.9, C27), 17.4 (C25), 19.4 (C6), 19.4 (C26), 22.0 (C16) 24.0 (C30), 25.0 (C15), 26.5 (*d*, ^2^*J*_C–F_ 21.5, C11), 26.9 (C19), 27.3 (C2), 27.9 (C23), 30.7 (C20), 33.3 (C29), 33.6 (C21), 37.1(C22), 37.6 (C10), 37.8 (C7), 38.8 (C1), 39.0 (C4), 39.5 (C17), 43.6 (C8), 45.1 (*d*, ^2^*J*_C–F_ 18.9, C13), 45.5 (C18), 48.8 (*d*, ^3^*J*_C–F_ 2.5, C(9), 55.9 (C5), 78.9 (C3), 90.9 (*d*, ^3^*J*_C–F_ 7.3, C(14), 100.1 (*d*, ^1^*J*_C–F_ 179.5, C12), 178.6 (C28). δ_F_ (CDCl_3_, 376 MHz) −184.6 (*ddd*, ^1^*J*_C–F_ 106, 53, 24). *m*/*z* HR–MS [ESI^+^] calculated for C_30_H_48_FO_3_[(*M*+H)^+^] 475.3587, found 475.3582.

## 5. Refinement

Crystal data, data collection and structure refinement details are summarized in Table 3 ▸. Hydrogen atoms were placed in calculated positions and refined using idealized geometries, except H9 in compound (**2**), which was allowed to refine freely. Hydrogen isotropic atomic displacement parameters were constrained to ride with the parent atom with an appropriate multiplier for the hybridization. Atomic displacement parameters for C1*B*–C7*B* in compound (**1**) were restrained with a strong isotropic atomic displacement parameter restraint in order to refine chemically sensible atomic displacement parameters.

## Supplementary Material

Crystal structure: contains datablock(s) 1, 2. DOI: 10.1107/S2056989024006480/yy2009sup1.cif

Structure factors: contains datablock(s) 1. DOI: 10.1107/S2056989024006480/yy20091sup2.hkl

Supporting information file. DOI: 10.1107/S2056989024006480/yy20091sup4.mol

Structure factors: contains datablock(s) 2. DOI: 10.1107/S2056989024006480/yy20092sup3.hkl

Supporting information file. DOI: 10.1107/S2056989024006480/yy20092sup5.mol

CCDC references: 2367298, 2367297

Additional supporting information:  crystallographic information; 3D view; checkCIF report

## Figures and Tables

**Table 1:** Hydrogen-bond geometry (Å, °) for **1**[Chem scheme1]

*D*—H⋯*A*	*D*—H	H⋯*A*	*D*⋯*A*	*D*—H⋯*A*
O13—H13⋯O3*A*	0.82	1.93	2.720 (8)	161
O14—H14⋯O3*D*^i^	0.82	1.94	2.714 (9)	157
O3*A*—H3*A*⋯O3*C*^ii^	0.82	2.03	2.819 (8)	163
O3*D*—H3*D*⋯O3*B*^iii^	0.82	2.01	2.816 (8)	166
O3*B*—H3*B*⋯O14^iv^	0.82	2.01	2.715 (8)	144
O3*C*—H3*C*⋯O13^ii^	0.82	2.07	2.708 (9)	135

Symmetry codes: (i) 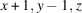; (ii) 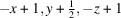; (iii) 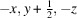; (iv) 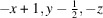.

**Table 2:** Hydrogen-bond geometry (Å, °) for **2**[Chem scheme1]

*D*—H⋯*A*	*D*—H	H⋯*A*	*D*⋯*A*	*D*—H⋯*A*
O3—H3*A*⋯O3^i^	0.82	2.12	2.784 (10)	138
O3—H3*B*⋯O4^ii^	0.83	1.94	2.695 (6)	152
O4—H4⋯O3^i^	0.82	1.96	2.695 (6)	149

Symmetry codes: (i) 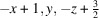; (ii) 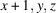.

**Table 3:** Experimental details

	**1**	**2**
Crystal data		
Chemical formula	2C_30_H_47_FO_3_·CH_4_O	2C_30_H_47_FO_3_·0CH_4_O
*M*_r_	981.39	981.39
Crystal system, space group	Monoclinic, *P*2_1_	Orthorhombic, *C*222_1_
Temperature (K)	100	240
*a*, *b*, *c* (Å)	12.4983 (2), 7.17223 (12), 60.3774 (12)	6.6077 (2), 13.8730 (4), 59.326 (2)
α, β, γ (°)	90, 94.1762 (17), 90	90, 90, 90
*V* (Å^3^)	5397.90 (17)	5438.3 (3)
*Z*	4	4
Radiation type	Cu *K*α	Cu *K*α
μ (mm^−1^)	0.64	0.64
Crystal size (mm)	0.42 × 0.24 × 0.05	0.34 × 0.2 × 0.1

Data collection		
Diffractometer	XtaLAB AFC11 (RINC): Kappa single	XtaLAB AFC11 (RINC): Kappa single
Absorption correction	Multi-scan (*CrysAlis PRO*; Rigaku OD, 2020 ▸)	Multi-scan (*CrysAlis PRO*; Rigaku OD, 2020 ▸)
*T*_min_, *T*_max_	0.486, 1.000	0.907, 1.000
No. of measured, independent and observed [*I* > 2σ(*I*)] reflections	77709, 21225, 19142	24920, 5184, 4937
*R*_int_	0.074	0.059
(sin θ/λ)_max_ (Å^−1^)	0.630	0.613

Refinement		
*R*[*F*^2^ > 2σ(*F*^2^)], *wR*(*F*^2^), *S*	0.098, 0.277, 1.05	0.073, 0.203, 1.09
No. of reflections	21225	5184
No. of parameters	1292	328
No. of restraints	43	0
H-atom treatment	H-atom parameters constrained	H atoms treated by a mixture of independent and constrained refinement
Δρ_max_, Δρ_min_ (e Å^−3^)	0.76, −0.54	0.27, −0.29
Absolute structure	Flack *x* determined using 7081 quotients [(*I*^+^)-(*I*^-^)]/[(*I*^+^)+(*I*^-^)] (Parsons *et al.*, 2013 ▸)	Refined as an inversion twin
Absolute structure parameter	0.04 (10)	0.5 (5)

Computer programs: *CrysAlis PRO* (Rigaku OD, 2020 ▸), *SHELXS* (Sheldrick, 2008 ▸), *SHELXT* (Sheldrick, 2015*a* ▸), *SHELXL2019/3* (Sheldrick, 2015*b* ▸) and *OLEX2* (Dolomanov *et al.*, 2009 ▸).

## supplementary crystallographic information

### 12-α-Fluoro-3β-hydroxyolean-28,13β-olide methanol hemisolvate (1) Crystal data

**Table:**

2C_30_H_47_FO_3_·CH_4_O	*F*(000) = 2152
*M**_r_* = 981.39	*D*_x_ = 1.208 Mg m^−^^3^
Monoclinic, *P*2_1_	Cu *K*α radiation, λ = 1.54184 Å
*a* = 12.4983 (2) Å	Cell parameters from 13347 reflections
*b* = 7.17223 (12) Å	θ = 1.5–74.7°
*c* = 60.3774 (12) Å	µ = 0.64 mm^−^^1^
β = 94.1762 (17)°	*T* = 100 K
*V* = 5397.90 (17) Å^3^	Plate, clear colourless
*Z* = 4	0.42 × 0.24 × 0.05 mm

### 12-α-Fluoro-3β-hydroxyolean-28,13β-olide methanol hemisolvate (1) Data collection

**Table:**

XtaLAB AFC11 (RINC): Kappa single diffractometer	21225 independent reflections
Radiation source: Rotating-anode X-ray tube (dual wavelength)	19142 reflections with *I* > 2σ(*I*)
Detector resolution: 10.0000 pixels mm^-1^	*R*_int_ = 0.074
ω scans	θ_max_ = 76.2°, θ_min_ = 2.2°
Absorption correction: multi-scan (CrysAlisPro; Rigaku OD, 2020)	*h* = −12→15
*T*_min_ = 0.486, *T*_max_ = 1.000	*k* = −8→8
77709 measured reflections	*l* = −74→75

### 12-α-Fluoro-3β-hydroxyolean-28,13β-olide methanol hemisolvate (1) Refinement

**Table:**

Refinement on *F*^2^	Hydrogen site location: mixed
Least-squares matrix: full	H-atom parameters constrained
*R*[*F*^2^ > 2σ(*F*^2^)] = 0.098	*w* = 1/[σ^2^(*F*_o_^2^) + (0.1319*P*)^2^ + 16.8795*P*] where *P* = (*F*_o_^2^ + 2*F*_c_^2^)/3
*wR*(*F*^2^) = 0.277	(Δ/σ)_max_ < 0.001
*S* = 1.05	Δρ_max_ = 0.76 e Å^−^^3^
21225 reflections	Δρ_min_ = −0.54 e Å^−^^3^
1292 parameters	Absolute structure: Flack *x* determined using 7081 quotients [(*I*^+^)-(*I*^-^)]/[(*I*^+^)+(*I*^-^)] (Parsons *et al.*, 2013)
43 restraints	Absolute structure parameter: 0.04 (10)
Primary atom site location: dual	

### 12-α-Fluoro-3β-hydroxyolean-28,13β-olide methanol hemisolvate (1) Special details

**Table:**

Geometry. All esds (except the esd in the dihedral angle between two l.s. planes) are estimated using the full covariance matrix. The cell esds are taken into account individually in the estimation of esds in distances, angles and torsion angles; correlations between esds in cell parameters are only used when they are defined by crystal symmetry. An approximate (isotropic) treatment of cell esds is used for estimating esds involving l.s. planes.
Refinement. Data were streaked. this was the best crystal that could be found. Streaking has resulted in prolate atoms as not all intensity for all resflections could be accurately captured. ISOR restraints applied to refine more realistic thermal parameters for these atoms.Data collection: X-ray diffraction data for compounds (1) and (2) were collected using a dual wavelength Rigaku FR-X rotating anode diffractometer using CuKα (λ = 1.54184 Å) radiation, equipped with an AFC-11 4-circle kappa goniometer, VariMAX^TM^ microfocus optics, a Hypix-6000HE detector and an Oxford Cryosystems 800 plus nitrogen flow gas system, at temperatures of 240K and 100K, respectively. Data were collected and reduced using CrysAlisPro v40. Absorption correction was performed using empirical methods (SCALE3 ABSPACK) based upon symmetry-equivalent reflections combined with measurements at different azimuthal angles.Crystal structure determination and refinements: The crystal structure was solved and refined against all F^2^ values using the SHELX and Olex2 suite of programmes. All non-hydrogen atoms were refined anisotropically.

### 12-α-Fluoro-3β-hydroxyolean-28,13β-olide methanol hemisolvate (1) Fractional atomic coordinates and isotropic or equivalent isotropic displacement parameters (Å^2^)

**Table:**

	*x*	*y*	*z*	*U*_iso_*/*U*_eq_	
O13	0.2010 (5)	−0.0663 (9)	0.49918 (11)	0.0378 (14)	
H13	0.227588	0.036854	0.497586	0.057*	
C121	0.0913 (7)	−0.0603 (13)	0.49219 (19)	0.044 (2)	
H12E	0.082825	−0.030947	0.476307	0.066*	
H12F	0.055924	0.035958	0.500569	0.066*	
H12G	0.058565	−0.181743	0.494824	0.066*	
O14	0.6992 (5)	0.0744 (9)	0.00058 (11)	0.0399 (14)	
H14	0.723344	−0.031516	−0.000119	0.060*	
C122	0.5940 (8)	0.0669 (16)	0.0077 (2)	0.051 (3)	
H12H	0.548650	−0.008940	−0.002700	0.077*	
H12I	0.564632	0.193367	0.008223	0.077*	
H12J	0.595871	0.011038	0.022545	0.077*	
F1A	0.1075 (3)	0.4109 (7)	0.36114 (8)	0.0341 (11)	
O1A	0.3127 (4)	0.7268 (8)	0.34616 (9)	0.0267 (11)	
O2A	0.4107 (5)	0.8594 (9)	0.32041 (10)	0.0369 (14)	
O3A	0.2403 (5)	0.2994 (8)	0.49106 (9)	0.0317 (12)	
H3A	0.240270	0.400417	0.497400	0.047*	
C1A	0.1610 (6)	0.4831 (11)	0.43359 (13)	0.0265 (16)	
H1AA	0.105929	0.575476	0.428370	0.032*	
H1AB	0.140697	0.361522	0.426740	0.032*	
C2A	0.1616 (6)	0.4656 (12)	0.45857 (14)	0.0306 (17)	
H2AA	0.089791	0.424517	0.462534	0.037*	
H2AB	0.175915	0.589457	0.465376	0.037*	
C3A	0.2453 (7)	0.3281 (11)	0.46806 (13)	0.0285 (16)	
H3AA	0.228476	0.205527	0.460704	0.034*	
C4A	0.3606 (6)	0.3830 (11)	0.46205 (14)	0.0278 (16)	
C5A	0.3573 (6)	0.4112 (11)	0.43671 (13)	0.0259 (15)	
H5A	0.337445	0.285740	0.430447	0.031*	
C6A	0.4679 (6)	0.4520 (12)	0.42803 (14)	0.0316 (17)	
H6AA	0.487892	0.583111	0.431353	0.038*	
H6AB	0.522486	0.370588	0.435800	0.038*	
C7A	0.4670 (6)	0.4191 (12)	0.40325 (14)	0.0307 (17)	
H7AA	0.457738	0.284000	0.400373	0.037*	
H7AB	0.537702	0.455731	0.398246	0.037*	
C8A	0.3794 (5)	0.5250 (11)	0.38929 (12)	0.0237 (15)	
C9A	0.2697 (6)	0.5153 (10)	0.40019 (13)	0.0233 (15)	
H9A	0.245792	0.383288	0.397870	0.028*	
C10A	0.2711 (6)	0.5439 (11)	0.42576 (13)	0.0256 (15)	
C11A	0.1853 (6)	0.6295 (11)	0.38662 (13)	0.0261 (16)	
H11A	0.115943	0.614895	0.393353	0.031*	
H11B	0.205666	0.762688	0.387821	0.031*	
C12A	0.1692 (5)	0.5801 (11)	0.36244 (13)	0.0258 (15)	
H12A	0.127823	0.681624	0.354332	0.031*	
C13A	0.2720 (6)	0.5393 (11)	0.35072 (12)	0.0240 (15)	
C14A	0.3617 (5)	0.4344 (11)	0.36511 (12)	0.0242 (14)	
C15A	0.4662 (5)	0.4408 (12)	0.35280 (14)	0.0288 (16)	
H15A	0.501742	0.562443	0.355804	0.035*	
H15B	0.515275	0.342220	0.358917	0.035*	
C16A	0.4488 (6)	0.4144 (12)	0.32781 (13)	0.0293 (16)	
H16A	0.435998	0.280543	0.324643	0.035*	
H16B	0.515228	0.450779	0.320911	0.035*	
C17A	0.3544 (6)	0.5279 (11)	0.31694 (13)	0.0251 (15)	
C18A	0.2500 (6)	0.4707 (12)	0.32640 (13)	0.0269 (16)	
H18A	0.194202	0.556945	0.319500	0.032*	
C19A	0.2145 (6)	0.2734 (12)	0.31882 (14)	0.0321 (17)	
H19A	0.145128	0.242534	0.324842	0.039*	
H19B	0.268385	0.180999	0.324610	0.039*	
C20A	0.2029 (6)	0.2639 (14)	0.29321 (14)	0.0342 (18)	
C21A	0.3070 (7)	0.3285 (13)	0.28363 (14)	0.0326 (18)	
H21A	0.296016	0.331042	0.267224	0.039*	
H21B	0.364215	0.236664	0.287676	0.039*	
C22A	0.3446 (6)	0.5221 (11)	0.29183 (14)	0.0292 (16)	
H22A	0.415022	0.550831	0.286110	0.035*	
H22B	0.292621	0.617654	0.286084	0.035*	
C23A	0.4336 (8)	0.2169 (15)	0.46882 (15)	0.043 (2)	
H23A	0.420354	0.177366	0.483909	0.064*	
H23B	0.508887	0.254161	0.468354	0.064*	
H23C	0.418252	0.113232	0.458494	0.064*	
C24A	0.4027 (7)	0.5495 (13)	0.47614 (14)	0.0350 (18)	
H24A	0.350924	0.652155	0.474564	0.052*	
H24B	0.471592	0.590423	0.471077	0.052*	
H24C	0.412285	0.512038	0.491768	0.052*	
C25A	0.2871 (7)	0.7524 (11)	0.43231 (13)	0.0308 (16)	
H25A	0.362371	0.787060	0.431251	0.046*	
H25B	0.267534	0.770859	0.447583	0.046*	
H25C	0.241369	0.830495	0.422225	0.046*	
C26A	0.4192 (6)	0.7291 (11)	0.38877 (13)	0.0264 (15)	
H26A	0.360022	0.810311	0.383276	0.040*	
H26B	0.477891	0.738527	0.378916	0.040*	
H26C	0.444880	0.767829	0.403795	0.040*	
C27A	0.3266 (7)	0.2250 (12)	0.36667 (13)	0.0315 (17)	
H27A	0.341944	0.180210	0.381906	0.047*	
H27B	0.366317	0.149600	0.356505	0.047*	
H27C	0.249471	0.214379	0.362579	0.047*	
C28A	0.3665 (7)	0.7229 (12)	0.32693 (13)	0.0306 (17)	
C29A	0.1072 (7)	0.3815 (17)	0.28368 (16)	0.045 (2)	
H29A	0.042280	0.343998	0.290686	0.068*	
H29B	0.096851	0.361537	0.267608	0.068*	
H29C	0.121719	0.513712	0.286675	0.068*	
C30A	0.1830 (7)	0.0579 (14)	0.28648 (16)	0.040 (2)	
H30A	0.243136	−0.018707	0.292519	0.060*	
H30B	0.176674	0.047674	0.270255	0.060*	
H30C	0.116462	0.014253	0.292418	0.060*	
F1D	−0.3048 (3)	0.5948 (7)	0.13742 (8)	0.0349 (11)	
O1D	−0.0917 (4)	0.2741 (7)	0.15326 (9)	0.0266 (11)	
O2D	0.0232 (5)	0.1409 (9)	0.17898 (11)	0.0374 (14)	
O3D	−0.2534 (5)	0.7097 (9)	0.00820 (10)	0.0362 (13)	
H3D	−0.262068	0.609859	0.001688	0.054*	
C1D	−0.2974 (6)	0.5220 (11)	0.06531 (14)	0.0295 (17)	
H1DA	−0.349438	0.429149	0.070177	0.035*	
H1DB	−0.312891	0.642607	0.072376	0.035*	
C2D	−0.3124 (6)	0.5434 (12)	0.04016 (14)	0.0308 (17)	
H2DA	−0.304470	0.419832	0.033149	0.037*	
H2DB	−0.386013	0.588606	0.036053	0.037*	
C3D	−0.2329 (7)	0.6769 (11)	0.03126 (14)	0.0299 (17)	
H3DA	−0.243996	0.798951	0.038761	0.036*	
C4D	−0.1149 (6)	0.6204 (11)	0.03755 (15)	0.0305 (17)	
C5D	−0.1010 (6)	0.5916 (10)	0.06285 (13)	0.0253 (15)	
H5D	−0.116113	0.716751	0.069222	0.030*	
C6D	0.0142 (6)	0.5471 (12)	0.07181 (14)	0.0307 (17)	
H6DA	0.030548	0.415354	0.068522	0.037*	
H6DB	0.064746	0.626617	0.064168	0.037*	
C7D	0.0302 (6)	0.5794 (11)	0.09662 (13)	0.0271 (15)	
H7DA	0.024084	0.714640	0.099562	0.033*	
H7DB	0.103775	0.540306	0.101786	0.033*	
C8D	−0.0501 (5)	0.4747 (11)	0.11020 (13)	0.0236 (15)	
C9D	−0.1665 (5)	0.4859 (10)	0.09895 (13)	0.0240 (15)	
H9D	−0.188648	0.617991	0.101285	0.029*	
C10D	−0.1811 (6)	0.4584 (11)	0.07331 (13)	0.0260 (15)	
C11D	−0.2445 (6)	0.3693 (12)	0.11224 (13)	0.0289 (16)	
H11C	−0.318165	0.383327	0.105219	0.035*	
H11D	−0.224676	0.235971	0.111367	0.035*	
C12D	−0.2441 (6)	0.4251 (12)	0.13639 (13)	0.0293 (16)	
H12D	−0.280414	0.325427	0.144668	0.035*	
C13D	−0.1348 (6)	0.4653 (11)	0.14831 (12)	0.0243 (15)	
C14D	−0.0531 (6)	0.5679 (11)	0.13456 (12)	0.0246 (15)	
C15D	0.0583 (6)	0.5583 (12)	0.14722 (14)	0.0300 (17)	
H15C	0.091157	0.436467	0.144074	0.036*	
H15D	0.104150	0.656592	0.141365	0.036*	
C16D	0.0587 (6)	0.5819 (12)	0.17250 (14)	0.0287 (16)	
H16C	0.050557	0.715799	0.175981	0.034*	
H16D	0.128815	0.540094	0.179424	0.034*	
C17D	−0.0317 (6)	0.4710 (11)	0.18275 (14)	0.0268 (15)	
C18D	−0.1415 (5)	0.5336 (11)	0.17248 (13)	0.0243 (15)	
H18D	−0.195259	0.451162	0.179089	0.029*	
C19D	−0.1681 (6)	0.7325 (11)	0.17957 (13)	0.0275 (16)	
H19C	−0.240375	0.767565	0.173089	0.033*	
H19D	−0.115533	0.820794	0.173950	0.033*	
C20D	−0.1650 (6)	0.7450 (11)	0.20514 (14)	0.0296 (16)	
C21D	−0.0584 (7)	0.6705 (12)	0.21573 (14)	0.0325 (17)	
H21C	−0.062096	0.667665	0.232046	0.039*	
H21D	−0.000509	0.758294	0.212413	0.039*	
C22D	−0.0286 (6)	0.4756 (12)	0.20785 (14)	0.0308 (17)	
H22C	0.044214	0.442462	0.214174	0.037*	
H22D	−0.079720	0.382909	0.213106	0.037*	
C23D	−0.0452 (8)	0.7871 (15)	0.03123 (15)	0.042 (2)	
H23D	−0.055791	0.891393	0.041335	0.063*	
H23E	−0.066178	0.825490	0.015946	0.063*	
H23F	0.030490	0.750437	0.032395	0.063*	
C24D	−0.0810 (7)	0.4530 (14)	0.02354 (14)	0.0372 (19)	
H24D	−0.081879	0.489838	0.007903	0.056*	
H24E	−0.131217	0.349606	0.025084	0.056*	
H24F	−0.008499	0.413319	0.028768	0.056*	
C25D	−0.1721 (7)	0.2518 (11)	0.06663 (13)	0.0301 (16)	
H25D	−0.096252	0.215915	0.067132	0.045*	
H25E	−0.204890	0.234149	0.051537	0.045*	
H25F	−0.209257	0.173948	0.076978	0.045*	
C26D	−0.0116 (6)	0.2686 (11)	0.11111 (13)	0.0278 (16)	
H26D	−0.069222	0.188812	0.115880	0.042*	
H26E	0.051406	0.257403	0.121692	0.042*	
H26F	0.007458	0.229791	0.096342	0.042*	
C27D	−0.0850 (7)	0.7756 (12)	0.13252 (13)	0.0328 (17)	
H27D	−0.045033	0.847217	0.144212	0.049*	
H27E	−0.162097	0.788553	0.134088	0.049*	
H27F	−0.067957	0.822756	0.117949	0.049*	
C28D	−0.0278 (6)	0.2774 (11)	0.17245 (13)	0.0279 (16)	
C29D	−0.2602 (7)	0.6442 (14)	0.21412 (14)	0.0355 (19)	
H29D	−0.326762	0.686069	0.206042	0.053*	
H29E	−0.263041	0.672348	0.229955	0.053*	
H29F	−0.252123	0.509433	0.212149	0.053*	
C30D	−0.1736 (8)	0.9511 (14)	0.21128 (15)	0.041 (2)	
H30D	−0.171714	0.963662	0.227468	0.062*	
H30E	−0.241217	1.001945	0.204603	0.062*	
H30F	−0.113307	1.019837	0.205707	0.062*	
F1B	0.4000 (5)	−0.3445 (7)	0.15133 (9)	0.0426 (13)	
O1B	0.5350 (4)	0.0789 (9)	0.16920 (10)	0.0318 (12)	
O2B	0.5938 (5)	0.2795 (10)	0.19574 (12)	0.0475 (17)	
O3B	0.2899 (5)	−0.0943 (8)	0.02089 (9)	0.0337 (13)	
H3B	0.265729	−0.193374	0.015961	0.051*	
C1B	0.4070 (6)	−0.2236 (10)	0.07830 (13)	0.0262 (15)	
H1BA	0.471590	−0.297242	0.083082	0.031*	
H1BB	0.344093	−0.288291	0.083764	0.031*	
C2B	0.3952 (6)	−0.2193 (10)	0.05335 (14)	0.0289 (16)	
H2BA	0.386400	−0.348379	0.047687	0.035*	
H2BB	0.461563	−0.167723	0.047738	0.035*	
C3B	0.2989 (6)	−0.1015 (10)	0.04436 (13)	0.0280 (16)	
H3BA	0.232604	−0.162934	0.049164	0.034*	
C4B	0.3019 (6)	0.0962 (10)	0.05378 (13)	0.0253 (15)	
C5B	0.3178 (6)	0.0870 (10)	0.07943 (13)	0.0233 (14)	
H5B	0.253696	0.018260	0.084151	0.028*	
C6B	0.3139 (6)	0.2769 (10)	0.09083 (13)	0.0265 (15)	
H6BA	0.380846	0.346115	0.088641	0.032*	
H6BB	0.253055	0.349983	0.083952	0.032*	
C7B	0.3011 (6)	0.2558 (10)	0.11548 (12)	0.0224 (14)	
H7BA	0.230370	0.198573	0.117491	0.027*	
H7BB	0.301438	0.381294	0.122312	0.027*	
C8B	0.3887 (6)	0.1368 (10)	0.12788 (13)	0.0236 (15)	
C9B	0.4061 (6)	−0.0484 (10)	0.11484 (13)	0.0255 (15)	
H9B	0.339040	−0.122187	0.116192	0.031*	
C10B	0.4167 (5)	−0.0285 (10)	0.08918 (13)	0.0226 (14)	
C11B	0.4952 (7)	−0.1645 (11)	0.12688 (14)	0.0295 (16)	
H11E	0.500908	−0.284678	0.118999	0.035*	
H11F	0.564244	−0.097927	0.126056	0.035*	
C12B	0.4786 (7)	−0.2038 (11)	0.15101 (14)	0.0317 (17)	
H12B	0.547192	−0.251923	0.158443	0.038*	
C13B	0.4392 (6)	−0.0409 (11)	0.16456 (13)	0.0252 (15)	
C14B	0.3523 (6)	0.0800 (10)	0.15178 (13)	0.0231 (14)	
C15B	0.3331 (6)	0.2558 (10)	0.16609 (13)	0.0266 (15)	
H15E	0.389587	0.348630	0.163608	0.032*	
H15F	0.263260	0.311602	0.160887	0.032*	
C16B	0.3329 (7)	0.2183 (11)	0.19086 (13)	0.0300 (16)	
H16E	0.265324	0.154448	0.193898	0.036*	
H16F	0.335078	0.338706	0.198900	0.036*	
C17B	0.4297 (6)	0.0961 (11)	0.19992 (13)	0.0273 (16)	
C18B	0.4210 (6)	−0.0978 (11)	0.18883 (13)	0.0246 (15)	
H18B	0.487769	−0.165379	0.194295	0.030*	
C19B	0.3286 (6)	−0.2131 (11)	0.19704 (13)	0.0263 (15)	
H19E	0.327764	−0.338306	0.190123	0.032*	
H19F	0.259351	−0.151350	0.192699	0.032*	
C20B	0.3427 (6)	−0.2323 (12)	0.22277 (13)	0.0278 (16)	
C21B	0.3531 (7)	−0.0361 (12)	0.23365 (13)	0.0306 (17)	
H21E	0.283559	0.029558	0.231190	0.037*	
H21F	0.368231	−0.051310	0.249873	0.037*	
C22B	0.4404 (7)	0.0844 (12)	0.22476 (14)	0.0336 (18)	
H22E	0.436411	0.211425	0.231059	0.040*	
H22F	0.511469	0.031569	0.229608	0.040*	
C23B	0.1903 (7)	0.1844 (13)	0.04754 (14)	0.0329 (18)	
H23G	0.169867	0.161643	0.031799	0.049*	
H23H	0.193739	0.319051	0.050287	0.049*	
H23I	0.136923	0.128314	0.056612	0.049*	
C24B	0.3866 (7)	0.2173 (12)	0.04298 (13)	0.0312 (17)	
H24G	0.457201	0.158172	0.045299	0.047*	
H24H	0.388954	0.341354	0.049794	0.047*	
H24I	0.367137	0.228871	0.027022	0.047*	
C25B	0.5278 (6)	0.0522 (12)	0.08405 (13)	0.0287 (16)	
H25G	0.542907	0.019434	0.068839	0.043*	
H25H	0.583576	−0.000157	0.094485	0.043*	
H25I	0.526735	0.188187	0.085611	0.043*	
C26B	0.4919 (6)	0.2590 (11)	0.12909 (14)	0.0280 (16)	
H26G	0.484922	0.359864	0.139845	0.042*	
H26H	0.501524	0.312387	0.114424	0.042*	
H26I	0.554173	0.181817	0.133761	0.042*	
C27B	0.2448 (6)	−0.0313 (11)	0.14986 (13)	0.0267 (15)	
H27G	0.199661	0.013538	0.137004	0.040*	
H27H	0.207150	−0.013183	0.163386	0.040*	
H27I	0.259924	−0.164241	0.148001	0.040*	
C28B	0.5274 (7)	0.1642 (13)	0.18911 (15)	0.0335 (18)	
C29B	0.4423 (7)	−0.3479 (12)	0.23034 (13)	0.0300 (16)	
H29G	0.441818	−0.374368	0.246254	0.045*	
H29H	0.507159	−0.277645	0.227527	0.045*	
H29I	0.441357	−0.465485	0.222053	0.045*	
C30B	0.2433 (7)	−0.3292 (14)	0.23056 (14)	0.0348 (18)	
H30G	0.236527	−0.453372	0.223832	0.052*	
H30H	0.179432	−0.255236	0.226075	0.052*	
H30I	0.250135	−0.341122	0.246772	0.052*	
F1C	0.8093 (5)	0.3451 (7)	0.34930 (9)	0.0401 (12)	
O1C	0.9292 (4)	−0.0814 (8)	0.33233 (9)	0.0290 (11)	
O2C	0.9693 (5)	−0.2869 (10)	0.30602 (11)	0.0443 (16)	
O3C	0.7735 (5)	0.1025 (8)	0.47955 (10)	0.0373 (14)	
H3C	0.744835	0.195684	0.484334	0.056*	
C1C	0.8581 (6)	0.2275 (10)	0.42259 (13)	0.0253 (15)	
H1CA	0.919843	0.300812	0.418014	0.030*	
H1CB	0.791765	0.291899	0.416804	0.030*	
C2C	0.8615 (6)	0.2244 (11)	0.44778 (14)	0.0301 (16)	
H2CA	0.857294	0.353652	0.453397	0.036*	
H2CB	0.930479	0.170208	0.453760	0.036*	
C3C	0.7681 (6)	0.1090 (10)	0.45604 (13)	0.0270 (16)	
H3CA	0.699455	0.172275	0.450846	0.032*	
C4C	0.7643 (6)	−0.0911 (11)	0.44686 (13)	0.0263 (15)	
C5C	0.7667 (6)	−0.0832 (10)	0.42118 (13)	0.0229 (14)	
H5C	0.700307	−0.013080	0.416039	0.028*	
C6C	0.7541 (6)	−0.2720 (11)	0.40983 (13)	0.0275 (16)	
H6CA	0.821691	−0.343396	0.412434	0.033*	
H6CB	0.696456	−0.342964	0.416448	0.033*	
C7C	0.7273 (6)	−0.2526 (10)	0.38509 (13)	0.0237 (14)	
H7CA	0.655491	−0.194615	0.382619	0.028*	
H7CB	0.723265	−0.378500	0.378372	0.028*	
C8C	0.8084 (6)	−0.1354 (10)	0.37313 (12)	0.0233 (15)	
C9C	0.8335 (5)	0.0512 (10)	0.38620 (13)	0.0222 (14)	
H9C	0.765800	0.125974	0.384537	0.027*	
C10C	0.8612 (5)	0.0321 (10)	0.41190 (13)	0.0231 (15)	
C11C	0.9166 (6)	0.1628 (11)	0.37427 (13)	0.0272 (16)	
H11G	0.929508	0.282486	0.382186	0.033*	
H11H	0.984973	0.092681	0.375259	0.033*	
C12C	0.8858 (6)	0.2035 (10)	0.35039 (14)	0.0270 (16)	
H12C	0.950788	0.249348	0.343318	0.032*	
C13C	0.8370 (6)	0.0418 (10)	0.33643 (13)	0.0255 (15)	
C14C	0.7566 (5)	−0.0762 (10)	0.34887 (12)	0.0219 (14)	
C15C	0.7265 (6)	−0.2518 (11)	0.33461 (13)	0.0293 (16)	
H15G	0.784122	−0.345718	0.337219	0.035*	
H15H	0.659812	−0.305978	0.339732	0.035*	
C16C	0.7103 (6)	−0.2154 (11)	0.31007 (14)	0.0284 (16)	
H16G	0.641649	−0.148342	0.306871	0.034*	
H16H	0.705509	−0.335800	0.302047	0.034*	
C17C	0.8045 (6)	−0.0963 (12)	0.30135 (13)	0.0280 (16)	
C18C	0.8039 (6)	0.0962 (11)	0.31209 (12)	0.0250 (15)	
H18C	0.868414	0.160301	0.306828	0.030*	
C19C	0.7089 (6)	0.2176 (10)	0.30336 (13)	0.0247 (15)	
H19G	0.715654	0.343444	0.310064	0.030*	
H19H	0.641037	0.161644	0.307639	0.030*	
C20C	0.7067 (6)	0.2333 (11)	0.27792 (13)	0.0260 (15)	
C21C	0.7064 (7)	0.0383 (11)	0.26743 (13)	0.0293 (16)	
H21G	0.638003	−0.024290	0.270158	0.035*	
H21H	0.709222	0.051822	0.251169	0.035*	
C22C	0.7990 (7)	−0.0860 (12)	0.27618 (14)	0.0318 (17)	
H22G	0.789006	−0.212866	0.269902	0.038*	
H22H	0.867275	−0.035611	0.271369	0.038*	
C23C	0.6580 (7)	−0.1792 (13)	0.45274 (14)	0.0340 (18)	
H23J	0.659617	−0.313559	0.449806	0.051*	
H23K	0.598490	−0.122312	0.443672	0.051*	
H23L	0.648082	−0.158100	0.468496	0.051*	
C24C	0.8561 (7)	−0.2110 (12)	0.45813 (13)	0.0296 (16)	
H24J	0.844558	−0.226863	0.473897	0.044*	
H24K	0.924979	−0.148423	0.456669	0.044*	
H24L	0.856925	−0.333431	0.450937	0.044*	
C25C	0.9747 (6)	−0.0491 (12)	0.41754 (13)	0.0290 (16)	
H25J	1.001577	−0.007296	0.432367	0.043*	
H25K	1.023111	−0.005902	0.406562	0.043*	
H25L	0.971216	−0.185635	0.417265	0.043*	
C26C	0.9098 (6)	−0.2591 (11)	0.37235 (13)	0.0272 (15)	
H26J	0.971013	−0.181550	0.368917	0.041*	
H26K	0.897363	−0.354685	0.360853	0.041*	
H26L	0.925238	−0.319387	0.386804	0.041*	
C27C	0.6521 (6)	0.0365 (11)	0.35039 (13)	0.0251 (15)	
H27J	0.600506	0.001658	0.338084	0.038*	
H27K	0.667903	0.170055	0.349530	0.038*	
H27L	0.621474	0.009434	0.364534	0.038*	
C28C	0.9096 (7)	−0.1687 (13)	0.31237 (13)	0.0317 (18)	
C29C	0.8062 (7)	0.3414 (12)	0.27058 (14)	0.0314 (17)	
H29J	0.809010	0.464990	0.277527	0.047*	
H29K	0.800526	0.355238	0.254388	0.047*	
H29L	0.871625	0.272153	0.275200	0.047*	
C30C	0.6067 (7)	0.3386 (13)	0.26943 (14)	0.0323 (17)	
H30J	0.609027	0.465300	0.275523	0.048*	
H30K	0.543020	0.273955	0.274098	0.048*	
H30L	0.603424	0.344592	0.253177	0.048*	

### 12-α-Fluoro-3β-hydroxyolean-28,13β-olide methanol hemisolvate (1) Atomic displacement parameters (Å^2^)

**Table:**

	*U*^11^	*U*^22^	*U*^33^	*U*^12^	*U*^13^	*U*^23^
O13	0.035 (3)	0.020 (3)	0.058 (4)	−0.001 (2)	0.005 (3)	0.007 (3)
C121	0.033 (4)	0.019 (4)	0.080 (7)	−0.004 (3)	−0.002 (4)	0.007 (4)
O14	0.037 (3)	0.021 (3)	0.064 (4)	0.007 (3)	0.020 (3)	0.006 (3)
C122	0.042 (5)	0.037 (6)	0.078 (7)	0.000 (4)	0.027 (5)	0.004 (5)
F1A	0.018 (2)	0.035 (3)	0.051 (3)	−0.0109 (19)	0.0092 (18)	−0.006 (2)
O1A	0.030 (3)	0.020 (3)	0.032 (3)	−0.001 (2)	0.010 (2)	0.000 (2)
O2A	0.044 (3)	0.027 (3)	0.040 (3)	−0.011 (3)	0.010 (3)	0.002 (2)
O3A	0.040 (3)	0.016 (3)	0.040 (3)	−0.001 (2)	0.009 (2)	0.000 (2)
C1A	0.015 (3)	0.021 (4)	0.044 (4)	0.000 (3)	0.009 (3)	−0.002 (3)
C2A	0.019 (3)	0.024 (4)	0.049 (5)	0.002 (3)	0.007 (3)	−0.002 (3)
C3A	0.033 (4)	0.017 (4)	0.036 (4)	0.004 (3)	0.006 (3)	−0.002 (3)
C4A	0.022 (4)	0.019 (4)	0.044 (4)	0.001 (3)	0.005 (3)	−0.003 (3)
C5A	0.016 (3)	0.016 (4)	0.047 (4)	0.002 (3)	0.006 (3)	−0.004 (3)
C6A	0.023 (4)	0.027 (4)	0.044 (5)	0.003 (3)	0.005 (3)	0.006 (3)
C7A	0.021 (4)	0.025 (4)	0.047 (5)	0.007 (3)	0.007 (3)	0.004 (3)
C8A	0.011 (3)	0.027 (4)	0.034 (4)	0.002 (3)	0.005 (3)	0.001 (3)
C9A	0.017 (3)	0.012 (3)	0.042 (4)	0.003 (2)	0.008 (3)	0.001 (3)
C10A	0.022 (3)	0.019 (4)	0.036 (4)	−0.003 (3)	0.006 (3)	0.002 (3)
C11A	0.020 (3)	0.018 (4)	0.041 (4)	0.008 (3)	0.011 (3)	0.004 (3)
C12A	0.013 (3)	0.019 (4)	0.046 (4)	−0.003 (3)	0.008 (3)	0.003 (3)
C13A	0.021 (3)	0.019 (4)	0.033 (4)	−0.001 (3)	0.009 (3)	0.003 (3)
C14A	0.017 (3)	0.023 (4)	0.033 (4)	0.000 (3)	0.005 (3)	0.002 (3)
C15A	0.012 (3)	0.025 (4)	0.051 (5)	0.005 (3)	0.008 (3)	0.001 (3)
C16A	0.019 (3)	0.025 (4)	0.045 (4)	0.011 (3)	0.011 (3)	0.004 (3)
C17A	0.017 (3)	0.023 (4)	0.036 (4)	−0.003 (3)	0.005 (3)	−0.001 (3)
C18A	0.020 (3)	0.029 (4)	0.033 (4)	0.003 (3)	0.006 (3)	0.002 (3)
C19A	0.025 (4)	0.027 (4)	0.045 (5)	−0.003 (3)	0.007 (3)	−0.004 (3)
C20A	0.024 (4)	0.038 (5)	0.041 (5)	−0.001 (3)	0.007 (3)	−0.009 (4)
C21A	0.035 (4)	0.032 (5)	0.032 (4)	0.004 (3)	0.007 (3)	−0.005 (3)
C22A	0.028 (4)	0.017 (4)	0.043 (4)	−0.007 (3)	0.011 (3)	0.000 (3)
C23A	0.051 (6)	0.042 (5)	0.034 (4)	0.011 (4)	0.003 (4)	0.007 (4)
C24A	0.033 (4)	0.033 (5)	0.039 (4)	−0.003 (4)	0.005 (3)	−0.003 (4)
C25A	0.036 (4)	0.020 (4)	0.037 (4)	−0.003 (3)	0.007 (3)	−0.002 (3)
C26A	0.022 (3)	0.020 (4)	0.039 (4)	−0.004 (3)	0.009 (3)	−0.001 (3)
C27A	0.036 (4)	0.028 (4)	0.031 (4)	0.009 (3)	0.006 (3)	0.000 (3)
C28A	0.036 (4)	0.031 (4)	0.025 (4)	−0.004 (3)	0.007 (3)	0.002 (3)
C29A	0.026 (4)	0.067 (7)	0.042 (5)	0.015 (4)	0.000 (3)	−0.012 (5)
C30A	0.038 (5)	0.035 (5)	0.049 (5)	−0.009 (4)	0.009 (4)	−0.012 (4)
F1D	0.019 (2)	0.036 (3)	0.051 (3)	0.0130 (19)	0.0071 (18)	−0.004 (2)
O1D	0.027 (3)	0.013 (2)	0.041 (3)	0.000 (2)	0.006 (2)	0.002 (2)
O2D	0.039 (3)	0.028 (3)	0.046 (3)	0.009 (3)	0.008 (3)	0.003 (3)
O3D	0.047 (4)	0.024 (3)	0.038 (3)	0.002 (3)	0.007 (3)	−0.003 (2)
C1D	0.018 (3)	0.022 (4)	0.050 (5)	0.000 (3)	0.008 (3)	−0.004 (3)
C2D	0.024 (4)	0.020 (4)	0.049 (5)	0.002 (3)	0.004 (3)	0.001 (3)
C3D	0.036 (4)	0.018 (4)	0.036 (4)	−0.003 (3)	0.008 (3)	−0.006 (3)
C4D	0.027 (4)	0.018 (4)	0.048 (5)	−0.004 (3)	0.010 (3)	−0.005 (3)
C5D	0.020 (3)	0.013 (3)	0.044 (4)	−0.008 (3)	0.013 (3)	−0.003 (3)
C6D	0.019 (3)	0.029 (4)	0.045 (5)	0.000 (3)	0.010 (3)	0.002 (3)
C7D	0.019 (3)	0.019 (4)	0.045 (4)	0.001 (3)	0.009 (3)	0.001 (3)
C8D	0.016 (3)	0.019 (4)	0.037 (4)	0.000 (3)	0.008 (3)	0.001 (3)
C9D	0.014 (3)	0.018 (4)	0.041 (4)	0.000 (3)	0.010 (3)	−0.001 (3)
C10D	0.020 (3)	0.019 (4)	0.040 (4)	0.003 (3)	0.007 (3)	0.004 (3)
C11D	0.020 (4)	0.031 (4)	0.036 (4)	−0.007 (3)	0.003 (3)	0.004 (3)
C12D	0.015 (3)	0.032 (4)	0.041 (4)	0.005 (3)	0.007 (3)	0.007 (3)
C13D	0.020 (3)	0.021 (4)	0.031 (4)	−0.001 (3)	0.005 (3)	0.004 (3)
C14D	0.019 (3)	0.023 (4)	0.032 (4)	−0.006 (3)	0.007 (3)	−0.001 (3)
C15D	0.013 (3)	0.024 (4)	0.053 (5)	−0.003 (3)	0.006 (3)	−0.001 (3)
C16D	0.017 (3)	0.024 (4)	0.046 (5)	−0.006 (3)	0.005 (3)	−0.001 (3)
C17D	0.020 (3)	0.020 (4)	0.041 (4)	0.001 (3)	0.006 (3)	−0.001 (3)
C18D	0.015 (3)	0.022 (4)	0.036 (4)	−0.005 (3)	0.005 (3)	0.002 (3)
C19D	0.021 (3)	0.017 (4)	0.045 (4)	−0.002 (3)	0.008 (3)	0.002 (3)
C20D	0.026 (4)	0.017 (4)	0.047 (5)	−0.002 (3)	0.010 (3)	−0.001 (3)
C21D	0.035 (4)	0.028 (4)	0.035 (4)	0.001 (3)	0.005 (3)	−0.005 (3)
C22D	0.023 (4)	0.022 (4)	0.047 (5)	0.005 (3)	0.001 (3)	−0.001 (3)
C23D	0.047 (5)	0.042 (5)	0.038 (5)	−0.008 (4)	0.011 (4)	0.008 (4)
C24D	0.035 (4)	0.046 (6)	0.031 (4)	0.007 (4)	0.007 (3)	−0.002 (4)
C25D	0.040 (4)	0.017 (4)	0.034 (4)	−0.005 (3)	0.006 (3)	−0.002 (3)
C26D	0.027 (4)	0.024 (4)	0.033 (4)	−0.004 (3)	0.005 (3)	−0.005 (3)
C27D	0.045 (5)	0.024 (4)	0.030 (4)	−0.003 (3)	0.009 (3)	0.000 (3)
C28D	0.033 (4)	0.020 (4)	0.032 (4)	0.004 (3)	0.008 (3)	0.003 (3)
C29D	0.029 (4)	0.045 (5)	0.034 (4)	−0.003 (4)	0.011 (3)	−0.001 (4)
C30D	0.050 (5)	0.037 (5)	0.038 (5)	0.010 (4)	0.013 (4)	−0.006 (4)
F1B	0.063 (3)	0.010 (2)	0.058 (3)	−0.008 (2)	0.026 (3)	−0.001 (2)
O1B	0.021 (3)	0.030 (3)	0.044 (3)	−0.007 (2)	0.004 (2)	0.004 (2)
O2B	0.039 (4)	0.038 (4)	0.065 (4)	−0.025 (3)	−0.003 (3)	0.002 (3)
O3B	0.047 (3)	0.015 (3)	0.039 (3)	0.001 (2)	0.007 (2)	−0.004 (2)
C1B	0.026 (3)	0.009 (3)	0.045 (4)	0.005 (3)	0.007 (3)	0.001 (3)
C2B	0.033 (4)	0.009 (3)	0.045 (4)	0.001 (3)	0.009 (3)	−0.003 (3)
C3B	0.032 (4)	0.011 (3)	0.041 (4)	0.000 (3)	0.005 (3)	−0.004 (3)
C4B	0.024 (3)	0.012 (3)	0.041 (4)	0.003 (3)	0.007 (3)	−0.002 (3)
C5B	0.019 (3)	0.007 (3)	0.045 (4)	0.001 (2)	0.007 (3)	−0.002 (3)
C6B	0.029 (4)	0.009 (3)	0.042 (4)	0.007 (3)	0.004 (3)	0.000 (3)
C7B	0.021 (3)	0.010 (3)	0.037 (4)	0.008 (2)	0.006 (3)	−0.002 (3)
C8B	0.025 (4)	0.010 (3)	0.036 (4)	0.006 (3)	0.004 (3)	0.002 (3)
C9B	0.024 (3)	0.015 (4)	0.039 (4)	0.003 (3)	0.006 (3)	−0.001 (3)
C10B	0.016 (3)	0.012 (3)	0.040 (4)	0.000 (3)	0.008 (3)	0.000 (3)
C11B	0.034 (4)	0.016 (4)	0.041 (4)	0.004 (3)	0.011 (3)	−0.001 (3)
C12B	0.033 (4)	0.017 (4)	0.046 (5)	0.006 (3)	0.009 (3)	0.001 (3)
C13B	0.024 (4)	0.016 (4)	0.036 (4)	−0.001 (3)	0.004 (3)	0.000 (3)
C14B	0.020 (3)	0.010 (3)	0.039 (4)	0.004 (3)	0.005 (3)	−0.002 (3)
C15B	0.027 (4)	0.011 (3)	0.041 (4)	0.001 (3)	0.003 (3)	−0.004 (3)
C16B	0.036 (4)	0.017 (4)	0.038 (4)	0.001 (3)	0.005 (3)	−0.004 (3)
C17B	0.029 (4)	0.014 (4)	0.039 (4)	−0.003 (3)	0.001 (3)	−0.001 (3)
C18B	0.022 (3)	0.017 (4)	0.035 (4)	0.006 (3)	0.005 (3)	0.000 (3)
C19B	0.025 (4)	0.019 (4)	0.034 (4)	−0.004 (3)	0.002 (3)	−0.002 (3)
C20B	0.030 (4)	0.023 (4)	0.032 (4)	−0.004 (3)	0.006 (3)	0.002 (3)
C21B	0.036 (4)	0.026 (4)	0.030 (4)	0.002 (3)	0.005 (3)	−0.002 (3)
C22B	0.040 (5)	0.015 (4)	0.046 (5)	−0.006 (3)	0.000 (3)	−0.003 (3)
C23B	0.036 (4)	0.030 (5)	0.033 (4)	0.007 (3)	0.002 (3)	−0.003 (3)
C24B	0.038 (4)	0.024 (4)	0.032 (4)	0.002 (3)	0.008 (3)	0.000 (3)
C25B	0.023 (4)	0.026 (4)	0.038 (4)	0.002 (3)	0.011 (3)	0.001 (3)
C26B	0.027 (4)	0.013 (4)	0.045 (4)	−0.002 (3)	0.009 (3)	0.002 (3)
C27B	0.026 (4)	0.017 (4)	0.038 (4)	−0.008 (3)	0.009 (3)	−0.003 (3)
C28B	0.027 (4)	0.030 (4)	0.042 (5)	−0.017 (3)	−0.003 (3)	0.006 (3)
C29B	0.030 (4)	0.026 (4)	0.034 (4)	0.004 (3)	0.001 (3)	0.003 (3)
C30B	0.031 (4)	0.041 (5)	0.034 (4)	−0.001 (4)	0.009 (3)	0.006 (3)
F1C	0.060 (3)	0.010 (2)	0.049 (3)	0.009 (2)	−0.005 (2)	−0.0015 (19)
O1C	0.025 (3)	0.028 (3)	0.035 (3)	0.002 (2)	0.009 (2)	0.004 (2)
O2C	0.045 (4)	0.039 (4)	0.051 (4)	0.028 (3)	0.016 (3)	0.001 (3)
O3C	0.057 (4)	0.018 (3)	0.038 (3)	0.001 (3)	0.010 (3)	−0.005 (2)
C1C	0.021 (3)	0.008 (3)	0.048 (4)	−0.007 (3)	0.006 (3)	−0.001 (3)
C2C	0.028 (4)	0.014 (4)	0.049 (5)	−0.004 (3)	0.006 (3)	−0.003 (3)
C3C	0.032 (4)	0.013 (4)	0.037 (4)	0.003 (3)	0.010 (3)	−0.002 (3)
C4C	0.027 (4)	0.014 (4)	0.038 (4)	−0.005 (3)	0.007 (3)	0.000 (3)
C5C	0.019 (3)	0.009 (3)	0.042 (4)	−0.001 (3)	0.005 (3)	−0.002 (3)
C6C	0.028 (4)	0.019 (4)	0.037 (4)	−0.006 (3)	0.011 (3)	−0.002 (3)
C7C	0.021 (3)	0.011 (3)	0.041 (4)	−0.003 (3)	0.008 (3)	−0.001 (3)
C8C	0.023 (4)	0.017 (4)	0.031 (4)	−0.004 (3)	0.008 (3)	0.000 (3)
C9C	0.016 (3)	0.008 (3)	0.043 (4)	−0.007 (2)	0.008 (3)	0.000 (3)
C10C	0.015 (3)	0.009 (3)	0.046 (4)	−0.005 (2)	0.009 (3)	−0.005 (3)
C11C	0.029 (4)	0.016 (4)	0.038 (4)	−0.009 (3)	0.009 (3)	0.000 (3)
C12C	0.015 (3)	0.013 (3)	0.054 (5)	−0.008 (3)	0.007 (3)	−0.003 (3)
C13C	0.025 (4)	0.014 (4)	0.038 (4)	−0.005 (3)	0.008 (3)	0.000 (3)
C14C	0.020 (3)	0.010 (3)	0.037 (4)	−0.001 (3)	0.008 (3)	−0.001 (3)
C15C	0.031 (4)	0.017 (4)	0.041 (4)	−0.010 (3)	0.006 (3)	−0.004 (3)
C16C	0.030 (4)	0.013 (3)	0.043 (4)	−0.007 (3)	0.009 (3)	−0.004 (3)
C17C	0.029 (4)	0.024 (4)	0.032 (4)	0.000 (3)	0.006 (3)	−0.001 (3)
C18C	0.022 (3)	0.024 (4)	0.029 (4)	−0.004 (3)	0.008 (3)	−0.004 (3)
C19C	0.021 (3)	0.010 (3)	0.044 (4)	0.000 (3)	0.009 (3)	−0.002 (3)
C20C	0.023 (4)	0.017 (4)	0.038 (4)	0.001 (3)	0.004 (3)	−0.002 (3)
C21C	0.038 (4)	0.023 (4)	0.027 (4)	−0.001 (3)	0.002 (3)	0.000 (3)
C22C	0.041 (4)	0.017 (4)	0.038 (4)	0.006 (3)	0.010 (3)	−0.001 (3)
C23C	0.035 (4)	0.032 (5)	0.037 (4)	−0.007 (3)	0.009 (3)	−0.003 (3)
C24C	0.036 (4)	0.022 (4)	0.031 (4)	0.002 (3)	0.005 (3)	0.001 (3)
C25C	0.027 (4)	0.026 (4)	0.034 (4)	0.003 (3)	0.000 (3)	0.001 (3)
C26C	0.030 (4)	0.018 (4)	0.034 (4)	0.001 (3)	0.005 (3)	0.003 (3)
C27C	0.019 (3)	0.017 (4)	0.040 (4)	0.005 (3)	0.004 (3)	−0.001 (3)
C28C	0.033 (4)	0.036 (5)	0.028 (4)	0.012 (3)	0.010 (3)	0.007 (3)
C29C	0.032 (4)	0.026 (4)	0.037 (4)	−0.004 (3)	0.007 (3)	0.005 (3)
C30C	0.028 (4)	0.033 (5)	0.036 (4)	0.008 (3)	−0.001 (3)	−0.003 (3)

### 12-α-Fluoro-3β-hydroxyolean-28,13β-olide methanol hemisolvate (1) Geometric parameters (Å, º)

**Table:**

O13—H13	0.8196	C29D—H29E	0.9800
O13—C121	1.406 (11)	C29D—H29F	0.9800
C121—H12E	0.9800	C30D—H30D	0.9800
C121—H12F	0.9800	C30D—H30E	0.9800
C121—H12G	0.9800	C30D—H30F	0.9800
O14—H14	0.8198	F1B—C12B	1.409 (10)
O14—C122	1.413 (11)	O1B—C13B	1.483 (9)
C122—H12H	0.9800	O1B—C28B	1.358 (11)
C122—H12I	0.9800	O2B—C28B	1.218 (10)
C122—H12J	0.9800	O3B—H3B	0.8198
F1A—C12A	1.437 (9)	O3B—C3B	1.414 (10)
O1A—C13A	1.470 (9)	C1B—H1BA	0.9900
O1A—C28A	1.384 (9)	C1B—H1BB	0.9900
O2A—C28A	1.204 (10)	C1B—C2B	1.503 (11)
O3A—H3A	0.8194	C1B—C10B	1.547 (10)
O3A—C3A	1.410 (10)	C2B—H2BA	0.9900
C1A—H1AA	0.9900	C2B—H2BB	0.9900
C1A—H1AB	0.9900	C2B—C3B	1.537 (11)
C1A—C2A	1.513 (11)	C3B—H3BA	1.0000
C1A—C10A	1.551 (10)	C3B—C4B	1.527 (10)
C2A—H2AA	0.9900	C4B—C5B	1.549 (11)
C2A—H2AB	0.9900	C4B—C23B	1.553 (11)
C2A—C3A	1.519 (11)	C4B—C24B	1.549 (11)
C3A—H3AA	1.0000	C5B—H5B	1.0000
C3A—C4A	1.562 (11)	C5B—C6B	1.528 (10)
C4A—C5A	1.540 (11)	C5B—C10B	1.567 (10)
C4A—C23A	1.538 (12)	C6B—H6BA	0.9900
C4A—C24A	1.536 (12)	C6B—H6BB	0.9900
C5A—H5A	1.0000	C6B—C7B	1.516 (11)
C5A—C6A	1.542 (10)	C7B—H7BA	0.9900
C5A—C10A	1.549 (10)	C7B—H7BB	0.9900
C6A—H6AA	0.9900	C7B—C8B	1.538 (9)
C6A—H6AB	0.9900	C8B—C9B	1.567 (10)
C6A—C7A	1.514 (12)	C8B—C14B	1.597 (10)
C7A—H7AA	0.9900	C8B—C26B	1.557 (10)
C7A—H7AB	0.9900	C9B—H9B	1.0000
C7A—C8A	1.533 (10)	C9B—C10B	1.571 (10)
C8A—C9A	1.565 (9)	C9B—C11B	1.531 (11)
C8A—C14A	1.599 (11)	C10B—C25B	1.556 (10)
C8A—C26A	1.548 (11)	C11B—H11E	0.9900
C9A—H9A	1.0000	C11B—H11F	0.9900
C9A—C10A	1.556 (11)	C11B—C12B	1.513 (12)
C9A—C11A	1.526 (10)	C12B—H12B	1.0000
C10A—C25A	1.556 (11)	C12B—C13B	1.528 (11)
C11A—H11A	0.9900	C13B—C14B	1.551 (10)
C11A—H11B	0.9900	C13B—C18B	1.554 (11)
C11A—C12A	1.501 (11)	C14B—C15B	1.557 (10)
C12A—H12A	1.0000	C14B—C27B	1.561 (10)
C12A—C13A	1.540 (9)	C15B—H15E	0.9900
C13A—C14A	1.561 (10)	C15B—H15F	0.9900
C13A—C18A	1.554 (11)	C15B—C16B	1.520 (11)
C14A—C15A	1.549 (9)	C16B—H16E	0.9900
C14A—C27A	1.570 (12)	C16B—H16F	0.9900
C15A—H15A	0.9900	C16B—C17B	1.560 (11)
C15A—H15B	0.9900	C17B—C18B	1.544 (10)
C15A—C16A	1.520 (11)	C17B—C22B	1.498 (12)
C16A—H16A	0.9900	C17B—C28B	1.507 (11)
C16A—H16B	0.9900	C18B—H18B	1.0000
C16A—C17A	1.540 (10)	C18B—C19B	1.532 (10)
C17A—C18A	1.519 (10)	C19B—H19E	0.9900
C17A—C22A	1.513 (11)	C19B—H19F	0.9900
C17A—C28A	1.527 (12)	C19B—C20B	1.557 (11)
C18A—H18A	1.0000	C20B—C21B	1.555 (11)
C18A—C19A	1.542 (12)	C20B—C29B	1.537 (11)
C19A—H19A	0.9900	C20B—C30B	1.527 (11)
C19A—H19B	0.9900	C21B—H21E	0.9900
C19A—C20A	1.544 (12)	C21B—H21F	0.9900
C20A—C21A	1.534 (12)	C21B—C22B	1.520 (12)
C20A—C29A	1.540 (12)	C22B—H22E	0.9900
C20A—C30A	1.547 (13)	C22B—H22F	0.9900
C21A—H21A	0.9900	C23B—H23G	0.9800
C21A—H21B	0.9900	C23B—H23H	0.9800
C21A—C22A	1.536 (12)	C23B—H23I	0.9800
C22A—H22A	0.9900	C24B—H24G	0.9800
C22A—H22B	0.9900	C24B—H24H	0.9800
C23A—H23A	0.9800	C24B—H24I	0.9800
C23A—H23B	0.9800	C25B—H25G	0.9800
C23A—H23C	0.9800	C25B—H25H	0.9800
C24A—H24A	0.9800	C25B—H25I	0.9800
C24A—H24B	0.9800	C26B—H26G	0.9800
C24A—H24C	0.9800	C26B—H26H	0.9800
C25A—H25A	0.9800	C26B—H26I	0.9800
C25A—H25B	0.9800	C27B—H27G	0.9800
C25A—H25C	0.9800	C27B—H27H	0.9800
C26A—H26A	0.9800	C27B—H27I	0.9800
C26A—H26B	0.9800	C29B—H29G	0.9800
C26A—H26C	0.9800	C29B—H29H	0.9800
C27A—H27A	0.9800	C29B—H29I	0.9800
C27A—H27B	0.9800	C30B—H30G	0.9800
C27A—H27C	0.9800	C30B—H30H	0.9800
C29A—H29A	0.9800	C30B—H30I	0.9800
C29A—H29B	0.9800	F1C—C12C	1.393 (9)
C29A—H29C	0.9800	O1C—C13C	1.488 (9)
C30A—H30A	0.9800	O1C—C28C	1.364 (10)
C30A—H30B	0.9800	O2C—C28C	1.210 (10)
C30A—H30C	0.9800	O3C—H3C	0.8203
F1D—C12D	1.438 (9)	O3C—C3C	1.417 (10)
O1D—C13D	1.495 (9)	C1C—H1CA	0.9900
O1D—C28D	1.359 (10)	C1C—H1CB	0.9900
O2D—C28D	1.217 (10)	C1C—C2C	1.518 (11)
O3D—H3D	0.8200	C1C—C10C	1.544 (10)
O3D—C3D	1.417 (10)	C2C—H2CA	0.9900
C1D—H1DA	0.9900	C2C—H2CB	0.9900
C1D—H1DB	0.9900	C2C—C3C	1.543 (10)
C1D—C2D	1.524 (12)	C3C—H3CA	1.0000
C1D—C10D	1.566 (10)	C3C—C4C	1.538 (11)
C2D—H2DA	0.9900	C4C—C5C	1.554 (11)
C2D—H2DB	0.9900	C4C—C23C	1.536 (11)
C2D—C3D	1.507 (11)	C4C—C24C	1.552 (11)
C3D—H3DA	1.0000	C5C—H5C	1.0000
C3D—C4D	1.550 (12)	C5C—C6C	1.521 (10)
C4D—C5D	1.539 (12)	C5C—C10C	1.577 (9)
C4D—C23D	1.543 (12)	C6C—H6CA	0.9900
C4D—C24D	1.545 (12)	C6C—H6CB	0.9900
C5D—H5D	1.0000	C6C—C7C	1.513 (11)
C5D—C6D	1.534 (11)	C7C—H7CA	0.9900
C5D—C10D	1.551 (10)	C7C—H7CB	0.9900
C6D—H6DA	0.9900	C7C—C8C	1.538 (9)
C6D—H6DB	0.9900	C8C—C9C	1.574 (10)
C6D—C7D	1.514 (11)	C8C—C14C	1.615 (10)
C7D—H7DA	0.9900	C8C—C26C	1.551 (10)
C7D—H7DB	0.9900	C9C—H9C	1.0000
C7D—C8D	1.537 (10)	C9C—C10C	1.571 (11)
C8D—C9D	1.562 (10)	C9C—C11C	1.532 (9)
C8D—C14D	1.618 (10)	C10C—C25C	1.549 (10)
C8D—C26D	1.554 (11)	C11C—H11G	0.9900
C9D—H9D	1.0000	C11C—H11H	0.9900
C9D—C10D	1.558 (11)	C11C—C12C	1.493 (12)
C9D—C11D	1.552 (10)	C12C—H12C	1.0000
C10D—C25D	1.542 (11)	C12C—C13C	1.535 (10)
C11D—H11C	0.9900	C13C—C14C	1.549 (9)
C11D—H11D	0.9900	C13C—C18C	1.548 (11)
C11D—C12D	1.512 (11)	C14C—C15C	1.556 (10)
C12D—H12D	1.0000	C14C—C27C	1.545 (9)
C12D—C13D	1.525 (10)	C15C—H15G	0.9900
C13D—C14D	1.548 (9)	C15C—H15H	0.9900
C13D—C18D	1.547 (11)	C15C—C16C	1.504 (11)
C14D—C15D	1.541 (10)	C16C—H16G	0.9900
C14D—C27D	1.544 (12)	C16C—H16H	0.9900
C15D—H15C	0.9900	C16C—C17C	1.576 (10)
C15D—H15D	0.9900	C17C—C18C	1.525 (11)
C15D—C16D	1.535 (12)	C17C—C22C	1.518 (11)
C16D—H16C	0.9900	C17C—C28C	1.521 (11)
C16D—H16D	0.9900	C18C—H18C	1.0000
C16D—C17D	1.547 (10)	C18C—C19C	1.534 (10)
C17D—C18D	1.532 (10)	C19C—H19G	0.9900
C17D—C22D	1.513 (12)	C19C—H19H	0.9900
C17D—C28D	1.524 (11)	C19C—C20C	1.538 (11)
C18D—H18D	1.0000	C20C—C21C	1.535 (11)
C18D—C19D	1.533 (11)	C20C—C29C	1.557 (10)
C19D—H19C	0.9900	C20C—C30C	1.518 (10)
C19D—H19D	0.9900	C21C—H21G	0.9900
C19D—C20D	1.544 (11)	C21C—H21H	0.9900
C20D—C21D	1.531 (12)	C21C—C22C	1.524 (11)
C20D—C29D	1.526 (11)	C22C—H22G	0.9900
C20D—C30D	1.530 (12)	C22C—H22H	0.9900
C21D—H21C	0.9900	C23C—H23J	0.9800
C21D—H21D	0.9900	C23C—H23K	0.9800
C21D—C22D	1.532 (11)	C23C—H23L	0.9800
C22D—H22C	0.9900	C24C—H24J	0.9800
C22D—H22D	0.9900	C24C—H24K	0.9800
C23D—H23D	0.9800	C24C—H24L	0.9800
C23D—H23E	0.9800	C25C—H25J	0.9800
C23D—H23F	0.9800	C25C—H25K	0.9800
C24D—H24D	0.9800	C25C—H25L	0.9800
C24D—H24E	0.9800	C26C—H26J	0.9800
C24D—H24F	0.9800	C26C—H26K	0.9800
C25D—H25D	0.9800	C26C—H26L	0.9800
C25D—H25E	0.9800	C27C—H27J	0.9800
C25D—H25F	0.9800	C27C—H27K	0.9800
C26D—H26D	0.9800	C27C—H27L	0.9800
C26D—H26E	0.9800	C29C—H29J	0.9800
C26D—H26F	0.9800	C29C—H29K	0.9800
C27D—H27D	0.9800	C29C—H29L	0.9800
C27D—H27E	0.9800	C30C—H30J	0.9800
C27D—H27F	0.9800	C30C—H30K	0.9800
C29D—H29D	0.9800	C30C—H30L	0.9800
			
C121—O13—H13	109.4	H29E—C29D—H29F	109.5
O13—C121—H12E	109.5	C20D—C30D—H30D	109.5
O13—C121—H12F	109.5	C20D—C30D—H30E	109.5
O13—C121—H12G	109.5	C20D—C30D—H30F	109.5
H12E—C121—H12F	109.5	H30D—C30D—H30E	109.5
H12E—C121—H12G	109.5	H30D—C30D—H30F	109.5
H12F—C121—H12G	109.5	H30E—C30D—H30F	109.5
C122—O14—H14	109.6	C28B—O1B—C13B	108.7 (6)
O14—C122—H12H	109.5	C3B—O3B—H3B	109.4
O14—C122—H12I	109.5	H1BA—C1B—H1BB	107.7
O14—C122—H12J	109.5	C2B—C1B—H1BA	108.8
H12H—C122—H12I	109.5	C2B—C1B—H1BB	108.8
H12H—C122—H12J	109.5	C2B—C1B—C10B	114.0 (6)
H12I—C122—H12J	109.5	C10B—C1B—H1BA	108.8
C28A—O1A—C13A	109.6 (6)	C10B—C1B—H1BB	108.8
C3A—O3A—H3A	109.4	C1B—C2B—H2BA	109.1
H1AA—C1A—H1AB	107.8	C1B—C2B—H2BB	109.1
C2A—C1A—H1AA	109.0	C1B—C2B—C3B	112.5 (6)
C2A—C1A—H1AB	109.0	H2BA—C2B—H2BB	107.8
C2A—C1A—C10A	112.8 (6)	C3B—C2B—H2BA	109.1
C10A—C1A—H1AA	109.0	C3B—C2B—H2BB	109.1
C10A—C1A—H1AB	109.0	O3B—C3B—C2B	112.1 (6)
C1A—C2A—H2AA	109.1	O3B—C3B—H3BA	107.4
C1A—C2A—H2AB	109.1	O3B—C3B—C4B	109.7 (6)
C1A—C2A—C3A	112.5 (6)	C2B—C3B—H3BA	107.4
H2AA—C2A—H2AB	107.8	C4B—C3B—C2B	112.4 (6)
C3A—C2A—H2AA	109.1	C4B—C3B—H3BA	107.4
C3A—C2A—H2AB	109.1	C3B—C4B—C5B	109.3 (6)
O3A—C3A—C2A	112.8 (6)	C3B—C4B—C23B	106.9 (6)
O3A—C3A—H3AA	106.6	C3B—C4B—C24B	111.3 (6)
O3A—C3A—C4A	111.9 (7)	C5B—C4B—C23B	107.9 (6)
C2A—C3A—H3AA	106.6	C5B—C4B—C24B	113.7 (6)
C2A—C3A—C4A	111.7 (7)	C24B—C4B—C23B	107.4 (7)
C4A—C3A—H3AA	106.6	C4B—C5B—H5B	105.0
C5A—C4A—C3A	107.8 (6)	C4B—C5B—C10B	116.0 (6)
C23A—C4A—C3A	106.4 (7)	C6B—C5B—C4B	114.0 (6)
C23A—C4A—C5A	109.7 (6)	C6B—C5B—H5B	105.0
C24A—C4A—C3A	110.6 (6)	C6B—C5B—C10B	110.6 (6)
C24A—C4A—C5A	115.6 (7)	C10B—C5B—H5B	105.0
C24A—C4A—C23A	106.4 (7)	C5B—C6B—H6BA	109.4
C4A—C5A—H5A	104.2	C5B—C6B—H6BB	109.4
C4A—C5A—C6A	113.7 (6)	H6BA—C6B—H6BB	108.0
C4A—C5A—C10A	118.2 (6)	C7B—C6B—C5B	111.2 (6)
C6A—C5A—H5A	104.2	C7B—C6B—H6BA	109.4
C6A—C5A—C10A	110.5 (6)	C7B—C6B—H6BB	109.4
C10A—C5A—H5A	104.2	C6B—C7B—H7BA	108.7
C5A—C6A—H6AA	109.4	C6B—C7B—H7BB	108.7
C5A—C6A—H6AB	109.4	C6B—C7B—C8B	114.2 (6)
H6AA—C6A—H6AB	108.0	H7BA—C7B—H7BB	107.6
C7A—C6A—C5A	111.4 (7)	C8B—C7B—H7BA	108.7
C7A—C6A—H6AA	109.4	C8B—C7B—H7BB	108.7
C7A—C6A—H6AB	109.4	C7B—C8B—C9B	110.3 (6)
C6A—C7A—H7AA	108.6	C7B—C8B—C14B	110.0 (6)
C6A—C7A—H7AB	108.6	C7B—C8B—C26B	105.6 (6)
C6A—C7A—C8A	114.8 (6)	C9B—C8B—C14B	107.3 (6)
H7AA—C7A—H7AB	107.5	C26B—C8B—C9B	110.8 (6)
C8A—C7A—H7AA	108.6	C26B—C8B—C14B	113.0 (6)
C8A—C7A—H7AB	108.6	C8B—C9B—H9B	105.0
C7A—C8A—C9A	111.1 (6)	C8B—C9B—C10B	116.3 (6)
C7A—C8A—C14A	110.3 (6)	C10B—C9B—H9B	105.0
C7A—C8A—C26A	105.2 (6)	C11B—C9B—C8B	110.2 (6)
C9A—C8A—C14A	107.4 (6)	C11B—C9B—H9B	105.0
C26A—C8A—C9A	110.1 (6)	C11B—C9B—C10B	114.0 (6)
C26A—C8A—C14A	112.9 (6)	C1B—C10B—C5B	106.5 (6)
C8A—C9A—H9A	104.2	C1B—C10B—C9B	109.1 (6)
C10A—C9A—C8A	117.7 (6)	C1B—C10B—C25B	107.2 (6)
C10A—C9A—H9A	104.2	C5B—C10B—C9B	107.3 (5)
C11A—C9A—C8A	110.1 (6)	C25B—C10B—C5B	114.8 (6)
C11A—C9A—H9A	104.2	C25B—C10B—C9B	111.7 (6)
C11A—C9A—C10A	114.8 (6)	C9B—C11B—H11E	108.7
C1A—C10A—C9A	108.6 (6)	C9B—C11B—H11F	108.7
C1A—C10A—C25A	107.0 (6)	H11E—C11B—H11F	107.6
C5A—C10A—C1A	107.6 (6)	C12B—C11B—C9B	114.3 (6)
C5A—C10A—C9A	107.5 (6)	C12B—C11B—H11E	108.7
C5A—C10A—C25A	114.2 (6)	C12B—C11B—H11F	108.7
C25A—C10A—C9A	111.8 (6)	F1B—C12B—C11B	107.0 (7)
C9A—C11A—H11A	108.3	F1B—C12B—H12B	108.8
C9A—C11A—H11B	108.3	F1B—C12B—C13B	106.8 (6)
H11A—C11A—H11B	107.4	C11B—C12B—H12B	108.8
C12A—C11A—C9A	115.8 (6)	C11B—C12B—C13B	116.4 (7)
C12A—C11A—H11A	108.3	C13B—C12B—H12B	108.8
C12A—C11A—H11B	108.3	O1B—C13B—C12B	104.9 (6)
F1A—C12A—C11A	106.6 (6)	O1B—C13B—C14B	107.3 (6)
F1A—C12A—H12A	109.4	O1B—C13B—C18B	98.4 (6)
F1A—C12A—C13A	106.1 (6)	C12B—C13B—C14B	113.7 (6)
C11A—C12A—H12A	109.4	C12B—C13B—C18B	112.3 (6)
C11A—C12A—C13A	115.7 (6)	C14B—C13B—C18B	118.1 (6)
C13A—C12A—H12A	109.4	C13B—C14B—C8B	111.0 (6)
O1A—C13A—C12A	102.9 (6)	C13B—C14B—C15B	107.9 (6)
O1A—C13A—C14A	107.5 (6)	C13B—C14B—C27B	108.6 (6)
O1A—C13A—C18A	98.8 (6)	C15B—C14B—C8B	111.1 (6)
C12A—C13A—C14A	115.2 (6)	C15B—C14B—C27B	106.7 (6)
C12A—C13A—C18A	113.4 (6)	C27B—C14B—C8B	111.3 (6)
C18A—C13A—C14A	116.3 (6)	C14B—C15B—H15E	108.6
C13A—C14A—C8A	111.0 (6)	C14B—C15B—H15F	108.6
C13A—C14A—C27A	107.6 (6)	H15E—C15B—H15F	107.6
C15A—C14A—C8A	111.1 (6)	C16B—C15B—C14B	114.5 (6)
C15A—C14A—C13A	108.4 (6)	C16B—C15B—H15E	108.6
C15A—C14A—C27A	107.8 (6)	C16B—C15B—H15F	108.6
C27A—C14A—C8A	110.8 (6)	C15B—C16B—H16E	109.0
C14A—C15A—H15A	108.7	C15B—C16B—H16F	109.0
C14A—C15A—H15B	108.7	C15B—C16B—C17B	112.8 (6)
H15A—C15A—H15B	107.6	H16E—C16B—H16F	107.8
C16A—C15A—C14A	114.1 (6)	C17B—C16B—H16E	109.0
C16A—C15A—H15A	108.7	C17B—C16B—H16F	109.0
C16A—C15A—H15B	108.7	C18B—C17B—C16B	109.0 (6)
C15A—C16A—H16A	108.8	C22B—C17B—C16B	113.1 (7)
C15A—C16A—H16B	108.8	C22B—C17B—C18B	112.5 (6)
C15A—C16A—C17A	114.0 (6)	C22B—C17B—C28B	115.8 (7)
H16A—C16A—H16B	107.7	C28B—C17B—C16B	107.3 (7)
C17A—C16A—H16A	108.8	C28B—C17B—C18B	98.0 (6)
C17A—C16A—H16B	108.8	C13B—C18B—H18B	105.2
C18A—C17A—C16A	110.5 (6)	C17B—C18B—C13B	99.3 (6)
C18A—C17A—C28A	99.3 (6)	C17B—C18B—H18B	105.2
C22A—C17A—C16A	114.6 (6)	C19B—C18B—C13B	127.8 (7)
C22A—C17A—C18A	111.1 (6)	C19B—C18B—C17B	112.2 (6)
C22A—C17A—C28A	114.8 (6)	C19B—C18B—H18B	105.2
C28A—C17A—C16A	105.3 (6)	C18B—C19B—H19E	109.7
C13A—C18A—H18A	105.5	C18B—C19B—H19F	109.7
C17A—C18A—C13A	99.9 (6)	C18B—C19B—C20B	109.7 (6)
C17A—C18A—H18A	105.5	H19E—C19B—H19F	108.2
C17A—C18A—C19A	111.8 (6)	C20B—C19B—H19E	109.7
C19A—C18A—C13A	126.8 (7)	C20B—C19B—H19F	109.7
C19A—C18A—H18A	105.5	C21B—C20B—C19B	109.9 (6)
C18A—C19A—H19A	109.7	C29B—C20B—C19B	112.0 (6)
C18A—C19A—H19B	109.7	C29B—C20B—C21B	108.8 (7)
C18A—C19A—C20A	110.0 (7)	C30B—C20B—C19B	108.1 (6)
H19A—C19A—H19B	108.2	C30B—C20B—C21B	109.1 (7)
C20A—C19A—H19A	109.7	C30B—C20B—C29B	108.8 (7)
C20A—C19A—H19B	109.7	C20B—C21B—H21E	108.8
C19A—C20A—C30A	107.9 (8)	C20B—C21B—H21F	108.8
C21A—C20A—C19A	110.1 (7)	H21E—C21B—H21F	107.7
C21A—C20A—C29A	110.5 (8)	C22B—C21B—C20B	113.9 (6)
C21A—C20A—C30A	108.3 (7)	C22B—C21B—H21E	108.8
C29A—C20A—C19A	111.4 (7)	C22B—C21B—H21F	108.8
C29A—C20A—C30A	108.6 (8)	C17B—C22B—C21B	111.8 (7)
C20A—C21A—H21A	108.8	C17B—C22B—H22E	109.3
C20A—C21A—H21B	108.9	C17B—C22B—H22F	109.3
C20A—C21A—C22A	113.6 (7)	C21B—C22B—H22E	109.3
H21A—C21A—H21B	107.7	C21B—C22B—H22F	109.3
C22A—C21A—H21A	108.9	H22E—C22B—H22F	107.9
C22A—C21A—H21B	108.9	C4B—C23B—H23G	109.5
C17A—C22A—C21A	110.4 (6)	C4B—C23B—H23H	109.5
C17A—C22A—H22A	109.6	C4B—C23B—H23I	109.5
C17A—C22A—H22B	109.6	H23G—C23B—H23H	109.5
C21A—C22A—H22A	109.6	H23G—C23B—H23I	109.5
C21A—C22A—H22B	109.6	H23H—C23B—H23I	109.5
H22A—C22A—H22B	108.1	C4B—C24B—H24G	109.5
C4A—C23A—H23A	109.5	C4B—C24B—H24H	109.5
C4A—C23A—H23B	109.5	C4B—C24B—H24I	109.5
C4A—C23A—H23C	109.5	H24G—C24B—H24H	109.5
H23A—C23A—H23B	109.5	H24G—C24B—H24I	109.5
H23A—C23A—H23C	109.5	H24H—C24B—H24I	109.5
H23B—C23A—H23C	109.5	C10B—C25B—H25G	109.5
C4A—C24A—H24A	109.5	C10B—C25B—H25H	109.5
C4A—C24A—H24B	109.5	C10B—C25B—H25I	109.5
C4A—C24A—H24C	109.5	H25G—C25B—H25H	109.5
H24A—C24A—H24B	109.5	H25G—C25B—H25I	109.5
H24A—C24A—H24C	109.5	H25H—C25B—H25I	109.5
H24B—C24A—H24C	109.5	C8B—C26B—H26G	109.5
C10A—C25A—H25A	109.5	C8B—C26B—H26H	109.5
C10A—C25A—H25B	109.5	C8B—C26B—H26I	109.5
C10A—C25A—H25C	109.5	H26G—C26B—H26H	109.5
H25A—C25A—H25B	109.5	H26G—C26B—H26I	109.5
H25A—C25A—H25C	109.5	H26H—C26B—H26I	109.5
H25B—C25A—H25C	109.5	C14B—C27B—H27G	109.5
C8A—C26A—H26A	109.5	C14B—C27B—H27H	109.5
C8A—C26A—H26B	109.5	C14B—C27B—H27I	109.5
C8A—C26A—H26C	109.5	H27G—C27B—H27H	109.5
H26A—C26A—H26B	109.5	H27G—C27B—H27I	109.5
H26A—C26A—H26C	109.5	H27H—C27B—H27I	109.5
H26B—C26A—H26C	109.5	O1B—C28B—C17B	110.4 (6)
C14A—C27A—H27A	109.5	O2B—C28B—O1B	120.4 (8)
C14A—C27A—H27B	109.5	O2B—C28B—C17B	129.2 (9)
C14A—C27A—H27C	109.5	C20B—C29B—H29G	109.5
H27A—C27A—H27B	109.5	C20B—C29B—H29H	109.5
H27A—C27A—H27C	109.5	C20B—C29B—H29I	109.5
H27B—C27A—H27C	109.5	H29G—C29B—H29H	109.5
O1A—C28A—C17A	108.0 (6)	H29G—C29B—H29I	109.5
O2A—C28A—O1A	121.4 (8)	H29H—C29B—H29I	109.5
O2A—C28A—C17A	130.6 (7)	C20B—C30B—H30G	109.5
C20A—C29A—H29A	109.5	C20B—C30B—H30H	109.5
C20A—C29A—H29B	109.5	C20B—C30B—H30I	109.5
C20A—C29A—H29C	109.5	H30G—C30B—H30H	109.5
H29A—C29A—H29B	109.5	H30G—C30B—H30I	109.5
H29A—C29A—H29C	109.5	H30H—C30B—H30I	109.5
H29B—C29A—H29C	109.5	C28C—O1C—C13C	109.1 (6)
C20A—C30A—H30A	109.5	C3C—O3C—H3C	109.6
C20A—C30A—H30B	109.5	H1CA—C1C—H1CB	107.7
C20A—C30A—H30C	109.5	C2C—C1C—H1CA	108.8
H30A—C30A—H30B	109.5	C2C—C1C—H1CB	108.8
H30A—C30A—H30C	109.5	C2C—C1C—C10C	113.9 (6)
H30B—C30A—H30C	109.5	C10C—C1C—H1CA	108.8
C28D—O1D—C13D	109.6 (6)	C10C—C1C—H1CB	108.8
C3D—O3D—H3D	109.5	C1C—C2C—H2CA	109.3
H1DA—C1D—H1DB	107.9	C1C—C2C—H2CB	109.3
C2D—C1D—H1DA	109.1	C1C—C2C—C3C	111.4 (6)
C2D—C1D—H1DB	109.1	H2CA—C2C—H2CB	108.0
C2D—C1D—C10D	112.4 (6)	C3C—C2C—H2CA	109.3
C10D—C1D—H1DA	109.1	C3C—C2C—H2CB	109.3
C10D—C1D—H1DB	109.1	O3C—C3C—C2C	111.1 (7)
C1D—C2D—H2DA	109.1	O3C—C3C—H3CA	107.9
C1D—C2D—H2DB	109.1	O3C—C3C—C4C	109.2 (6)
H2DA—C2D—H2DB	107.8	C2C—C3C—H3CA	107.9
C3D—C2D—C1D	112.5 (7)	C4C—C3C—C2C	112.8 (6)
C3D—C2D—H2DA	109.1	C4C—C3C—H3CA	107.9
C3D—C2D—H2DB	109.1	C3C—C4C—C5C	108.9 (6)
O3D—C3D—C2D	112.3 (7)	C3C—C4C—C24C	110.9 (6)
O3D—C3D—H3DA	106.2	C23C—C4C—C3C	107.8 (6)
O3D—C3D—C4D	112.6 (6)	C23C—C4C—C5C	109.0 (6)
C2D—C3D—H3DA	106.2	C23C—C4C—C24C	107.2 (7)
C2D—C3D—C4D	112.8 (7)	C24C—C4C—C5C	113.0 (6)
C4D—C3D—H3DA	106.2	C4C—C5C—H5C	104.6
C5D—C4D—C3D	108.4 (6)	C4C—C5C—C10C	116.3 (6)
C5D—C4D—C23D	108.9 (7)	C6C—C5C—C4C	114.1 (6)
C5D—C4D—C24D	115.0 (7)	C6C—C5C—H5C	104.6
C23D—C4D—C3D	106.3 (7)	C6C—C5C—C10C	111.2 (6)
C23D—C4D—C24D	106.6 (7)	C10C—C5C—H5C	104.6
C24D—C4D—C3D	111.3 (7)	C5C—C6C—H6CA	109.3
C4D—C5D—H5D	104.5	C5C—C6C—H6CB	109.3
C4D—C5D—C10D	117.2 (6)	H6CA—C6C—H6CB	107.9
C6D—C5D—C4D	114.4 (6)	C7C—C6C—C5C	111.8 (6)
C6D—C5D—H5D	104.5	C7C—C6C—H6CA	109.3
C6D—C5D—C10D	110.2 (6)	C7C—C6C—H6CB	109.3
C10D—C5D—H5D	104.5	C6C—C7C—H7CA	108.7
C5D—C6D—H6DA	109.3	C6C—C7C—H7CB	108.7
C5D—C6D—H6DB	109.3	C6C—C7C—C8C	114.0 (6)
H6DA—C6D—H6DB	107.9	H7CA—C7C—H7CB	107.6
C7D—C6D—C5D	111.7 (6)	C8C—C7C—H7CA	108.7
C7D—C6D—H6DA	109.3	C8C—C7C—H7CB	108.7
C7D—C6D—H6DB	109.3	C7C—C8C—C9C	110.1 (6)
C6D—C7D—H7DA	108.7	C7C—C8C—C14C	109.6 (6)
C6D—C7D—H7DB	108.7	C7C—C8C—C26C	105.7 (6)
C6D—C7D—C8D	114.1 (6)	C9C—C8C—C14C	106.4 (6)
H7DA—C7D—H7DB	107.6	C26C—C8C—C9C	111.7 (6)
C8D—C7D—H7DA	108.7	C26C—C8C—C14C	113.5 (6)
C8D—C7D—H7DB	108.7	C8C—C9C—H9C	105.7
C7D—C8D—C9D	111.5 (6)	C10C—C9C—C8C	116.3 (6)
C7D—C8D—C14D	110.0 (6)	C10C—C9C—H9C	105.7
C7D—C8D—C26D	105.7 (6)	C11C—C9C—C8C	109.0 (6)
C9D—C8D—C14D	106.9 (5)	C11C—C9C—H9C	105.7
C26D—C8D—C9D	109.9 (6)	C11C—C9C—C10C	113.7 (6)
C26D—C8D—C14D	112.9 (6)	C1C—C10C—C5C	106.6 (5)
C8D—C9D—H9D	104.3	C1C—C10C—C9C	108.8 (6)
C10D—C9D—C8D	117.7 (6)	C1C—C10C—C25C	107.4 (6)
C10D—C9D—H9D	104.3	C9C—C10C—C5C	106.2 (5)
C11D—C9D—C8D	110.4 (6)	C25C—C10C—C5C	115.0 (6)
C11D—C9D—H9D	104.3	C25C—C10C—C9C	112.5 (6)
C11D—C9D—C10D	114.4 (6)	C9C—C11C—H11G	108.6
C5D—C10D—C1D	107.9 (6)	C9C—C11C—H11H	108.6
C5D—C10D—C9D	107.1 (6)	H11G—C11C—H11H	107.5
C9D—C10D—C1D	108.0 (6)	C12C—C11C—C9C	114.9 (6)
C25D—C10D—C1D	106.5 (7)	C12C—C11C—H11G	108.6
C25D—C10D—C5D	115.1 (6)	C12C—C11C—H11H	108.6
C25D—C10D—C9D	112.1 (6)	F1C—C12C—C11C	108.4 (6)
C9D—C11D—H11C	108.9	F1C—C12C—H12C	108.5
C9D—C11D—H11D	108.9	F1C—C12C—C13C	106.3 (6)
H11C—C11D—H11D	107.7	C11C—C12C—H12C	108.5
C12D—C11D—C9D	113.4 (7)	C11C—C12C—C13C	116.5 (6)
C12D—C11D—H11C	108.9	C13C—C12C—H12C	108.5
C12D—C11D—H11D	108.9	O1C—C13C—C12C	105.0 (6)
F1D—C12D—C11D	107.5 (6)	O1C—C13C—C14C	107.1 (6)
F1D—C12D—H12D	108.8	O1C—C13C—C18C	98.6 (6)
F1D—C12D—C13D	106.1 (7)	C12C—C13C—C14C	113.1 (6)
C11D—C12D—H12D	108.8	C12C—C13C—C18C	113.5 (6)
C11D—C12D—C13D	116.5 (6)	C18C—C13C—C14C	117.4 (6)
C13D—C12D—H12D	108.8	C13C—C14C—C8C	110.6 (6)
O1D—C13D—C12D	102.6 (6)	C13C—C14C—C15C	108.2 (6)
O1D—C13D—C14D	107.5 (6)	C15C—C14C—C8C	110.7 (6)
O1D—C13D—C18D	98.4 (6)	C27C—C14C—C8C	111.7 (6)
C12D—C13D—C14D	116.1 (6)	C27C—C14C—C13C	108.9 (6)
C12D—C13D—C18D	113.4 (6)	C27C—C14C—C15C	106.6 (6)
C18D—C13D—C14D	116.0 (6)	C14C—C15C—H15G	108.6
C13D—C14D—C8D	110.6 (6)	C14C—C15C—H15H	108.6
C15D—C14D—C8D	110.6 (6)	H15G—C15C—H15H	107.6
C15D—C14D—C13D	108.7 (6)	C16C—C15C—C14C	114.5 (6)
C15D—C14D—C27D	107.5 (7)	C16C—C15C—H15G	108.6
C27D—C14D—C8D	110.4 (6)	C16C—C15C—H15H	108.6
C27D—C14D—C13D	109.0 (6)	C15C—C16C—H16G	109.2
C14D—C15D—H15C	108.5	C15C—C16C—H16H	109.2
C14D—C15D—H15D	108.5	C15C—C16C—C17C	111.9 (6)
H15C—C15D—H15D	107.5	H16G—C16C—H16H	107.9
C16D—C15D—C14D	115.2 (6)	C17C—C16C—H16G	109.2
C16D—C15D—H15C	108.5	C17C—C16C—H16H	109.2
C16D—C15D—H15D	108.5	C18C—C17C—C16C	108.8 (6)
C15D—C16D—H16C	109.0	C22C—C17C—C16C	112.3 (7)
C15D—C16D—H16D	109.0	C22C—C17C—C18C	112.4 (7)
C15D—C16D—C17D	113.1 (6)	C22C—C17C—C28C	115.4 (6)
H16C—C16D—H16D	107.8	C28C—C17C—C16C	108.2 (7)
C17D—C16D—H16C	109.0	C28C—C17C—C18C	98.9 (6)
C17D—C16D—H16D	109.0	C13C—C18C—H18C	104.6
C18D—C17D—C16D	110.2 (6)	C17C—C18C—C13C	99.6 (6)
C22D—C17D—C16D	115.1 (6)	C17C—C18C—H18C	104.6
C22D—C17D—C18D	110.8 (6)	C17C—C18C—C19C	113.3 (6)
C22D—C17D—C28D	115.4 (7)	C19C—C18C—C13C	128.0 (6)
C28D—C17D—C16D	105.0 (6)	C19C—C18C—H18C	104.6
C28D—C17D—C18D	99.0 (6)	C18C—C19C—H19G	109.7
C13D—C18D—H18D	105.9	C18C—C19C—H19H	109.7
C17D—C18D—C13D	100.4 (6)	C18C—C19C—C20C	110.0 (6)
C17D—C18D—H18D	105.9	H19G—C19C—H19H	108.2
C17D—C18D—C19D	111.5 (6)	C20C—C19C—H19G	109.7
C19D—C18D—C13D	125.9 (7)	C20C—C19C—H19H	109.7
C19D—C18D—H18D	105.9	C19C—C20C—C29C	111.3 (6)
C18D—C19D—H19C	109.7	C21C—C20C—C19C	110.2 (6)
C18D—C19D—H19D	109.7	C21C—C20C—C29C	108.3 (6)
C18D—C19D—C20D	110.0 (6)	C30C—C20C—C19C	109.2 (6)
H19C—C19D—H19D	108.2	C30C—C20C—C21C	109.7 (7)
C20D—C19D—H19C	109.7	C30C—C20C—C29C	108.1 (7)
C20D—C19D—H19D	109.7	C20C—C21C—H21G	108.7
C21D—C20D—C19D	110.8 (6)	C20C—C21C—H21H	108.7
C29D—C20D—C19D	111.4 (7)	H21G—C21C—H21H	107.6
C29D—C20D—C21D	111.4 (7)	C22C—C21C—C20C	114.3 (7)
C29D—C20D—C30D	107.5 (7)	C22C—C21C—H21G	108.7
C30D—C20D—C19D	107.5 (7)	C22C—C21C—H21H	108.7
C30D—C20D—C21D	108.1 (7)	C17C—C22C—C21C	110.7 (6)
C20D—C21D—H21C	108.7	C17C—C22C—H22G	109.5
C20D—C21D—H21D	108.7	C17C—C22C—H22H	109.5
C20D—C21D—C22D	114.3 (7)	C21C—C22C—H22G	109.5
H21C—C21D—H21D	107.6	C21C—C22C—H22H	109.5
C22D—C21D—H21C	108.7	H22G—C22C—H22H	108.1
C22D—C21D—H21D	108.7	C4C—C23C—H23J	109.5
C17D—C22D—C21D	110.0 (7)	C4C—C23C—H23K	109.5
C17D—C22D—H22C	109.7	C4C—C23C—H23L	109.5
C17D—C22D—H22D	109.7	H23J—C23C—H23K	109.5
C21D—C22D—H22C	109.7	H23J—C23C—H23L	109.5
C21D—C22D—H22D	109.7	H23K—C23C—H23L	109.5
H22C—C22D—H22D	108.2	C4C—C24C—H24J	109.5
C4D—C23D—H23D	109.5	C4C—C24C—H24K	109.5
C4D—C23D—H23E	109.5	C4C—C24C—H24L	109.5
C4D—C23D—H23F	109.5	H24J—C24C—H24K	109.5
H23D—C23D—H23E	109.5	H24J—C24C—H24L	109.5
H23D—C23D—H23F	109.5	H24K—C24C—H24L	109.5
H23E—C23D—H23F	109.5	C10C—C25C—H25J	109.5
C4D—C24D—H24D	109.5	C10C—C25C—H25K	109.5
C4D—C24D—H24E	109.5	C10C—C25C—H25L	109.5
C4D—C24D—H24F	109.5	H25J—C25C—H25K	109.5
H24D—C24D—H24E	109.5	H25J—C25C—H25L	109.5
H24D—C24D—H24F	109.5	H25K—C25C—H25L	109.5
H24E—C24D—H24F	109.5	C8C—C26C—H26J	109.5
C10D—C25D—H25D	109.5	C8C—C26C—H26K	109.5
C10D—C25D—H25E	109.5	C8C—C26C—H26L	109.5
C10D—C25D—H25F	109.5	H26J—C26C—H26K	109.5
H25D—C25D—H25E	109.5	H26J—C26C—H26L	109.5
H25D—C25D—H25F	109.5	H26K—C26C—H26L	109.5
H25E—C25D—H25F	109.5	C14C—C27C—H27J	109.5
C8D—C26D—H26D	109.5	C14C—C27C—H27K	109.5
C8D—C26D—H26E	109.5	C14C—C27C—H27L	109.5
C8D—C26D—H26F	109.5	H27J—C27C—H27K	109.5
H26D—C26D—H26E	109.5	H27J—C27C—H27L	109.5
H26D—C26D—H26F	109.5	H27K—C27C—H27L	109.5
H26E—C26D—H26F	109.5	O1C—C28C—C17C	108.9 (6)
C14D—C27D—H27D	109.5	O2C—C28C—O1C	121.8 (8)
C14D—C27D—H27E	109.5	O2C—C28C—C17C	129.3 (8)
C14D—C27D—H27F	109.5	C20C—C29C—H29J	109.5
H27D—C27D—H27E	109.5	C20C—C29C—H29K	109.5
H27D—C27D—H27F	109.5	C20C—C29C—H29L	109.5
H27E—C27D—H27F	109.5	H29J—C29C—H29K	109.5
O1D—C28D—C17D	109.2 (6)	H29J—C29C—H29L	109.5
O2D—C28D—O1D	121.6 (7)	H29K—C29C—H29L	109.5
O2D—C28D—C17D	129.2 (7)	C20C—C30C—H30J	109.5
C20D—C29D—H29D	109.5	C20C—C30C—H30K	109.5
C20D—C29D—H29E	109.5	C20C—C30C—H30L	109.5
C20D—C29D—H29F	109.5	H30J—C30C—H30K	109.5
H29D—C29D—H29E	109.5	H30J—C30C—H30L	109.5
H29D—C29D—H29F	109.5	H30K—C30C—H30L	109.5
			
F1A—C12A—C13A—O1A	162.2 (6)	F1B—C12B—C13B—O1B	164.5 (6)
F1A—C12A—C13A—C14A	−81.1 (8)	F1B—C12B—C13B—C14B	−78.7 (8)
F1A—C12A—C13A—C18A	56.5 (8)	F1B—C12B—C13B—C18B	58.7 (8)
O1A—C13A—C14A—C8A	67.9 (7)	O1B—C13B—C14B—C8B	66.1 (7)
O1A—C13A—C14A—C15A	−54.4 (7)	O1B—C13B—C14B—C15B	−55.9 (7)
O1A—C13A—C14A—C27A	−170.8 (5)	O1B—C13B—C14B—C27B	−171.2 (6)
O1A—C13A—C18A—C17A	47.2 (6)	O1B—C13B—C18B—C17B	47.7 (6)
O1A—C13A—C18A—C19A	174.2 (7)	O1B—C13B—C18B—C19B	175.3 (7)
O3A—C3A—C4A—C5A	−179.9 (6)	O3B—C3B—C4B—C5B	−177.0 (6)
O3A—C3A—C4A—C23A	62.4 (8)	O3B—C3B—C4B—C23B	66.4 (8)
O3A—C3A—C4A—C24A	−52.7 (9)	O3B—C3B—C4B—C24B	−50.5 (8)
C1A—C2A—C3A—O3A	−175.0 (6)	C1B—C2B—C3B—O3B	178.5 (6)
C1A—C2A—C3A—C4A	57.8 (9)	C1B—C2B—C3B—C4B	54.3 (9)
C2A—C1A—C10A—C5A	51.6 (8)	C2B—C1B—C10B—C5B	53.6 (8)
C2A—C1A—C10A—C9A	167.6 (6)	C2B—C1B—C10B—C9B	169.2 (6)
C2A—C1A—C10A—C25A	−71.6 (8)	C2B—C1B—C10B—C25B	−69.7 (8)
C2A—C3A—C4A—C5A	−52.3 (8)	C2B—C3B—C4B—C5B	−51.5 (8)
C2A—C3A—C4A—C23A	−170.0 (7)	C2B—C3B—C4B—C23B	−168.1 (7)
C2A—C3A—C4A—C24A	74.9 (9)	C2B—C3B—C4B—C24B	75.0 (8)
C3A—C4A—C5A—C6A	−175.7 (7)	C3B—C4B—C5B—C6B	−175.8 (6)
C3A—C4A—C5A—C10A	52.2 (9)	C3B—C4B—C5B—C10B	53.9 (8)
C4A—C5A—C6A—C7A	162.3 (7)	C4B—C5B—C6B—C7B	165.3 (6)
C4A—C5A—C10A—C1A	−51.6 (9)	C4B—C5B—C10B—C1B	−53.6 (7)
C4A—C5A—C10A—C9A	−168.4 (6)	C4B—C5B—C10B—C9B	−170.3 (6)
C4A—C5A—C10A—C25A	67.0 (8)	C4B—C5B—C10B—C25B	64.8 (8)
C5A—C6A—C7A—C8A	54.2 (9)	C5B—C6B—C7B—C8B	56.5 (8)
C6A—C5A—C10A—C1A	174.9 (6)	C6B—C5B—C10B—C1B	174.5 (6)
C6A—C5A—C10A—C9A	58.1 (8)	C6B—C5B—C10B—C9B	57.8 (7)
C6A—C5A—C10A—C25A	−66.5 (8)	C6B—C5B—C10B—C25B	−67.0 (8)
C6A—C7A—C8A—C9A	−43.3 (9)	C6B—C7B—C8B—C9B	−47.4 (8)
C6A—C7A—C8A—C14A	−162.3 (7)	C6B—C7B—C8B—C14B	−165.5 (6)
C6A—C7A—C8A—C26A	75.7 (8)	C6B—C7B—C8B—C26B	72.3 (8)
C7A—C8A—C9A—C10A	43.1 (9)	C7B—C8B—C9B—C10B	46.8 (8)
C7A—C8A—C9A—C11A	177.2 (7)	C7B—C8B—C9B—C11B	178.6 (6)
C7A—C8A—C14A—C13A	179.5 (6)	C7B—C8B—C14B—C13B	179.8 (6)
C7A—C8A—C14A—C15A	−59.8 (8)	C7B—C8B—C14B—C15B	−60.2 (8)
C7A—C8A—C14A—C27A	60.0 (8)	C7B—C8B—C14B—C27B	58.7 (7)
C8A—C9A—C10A—C1A	−166.7 (6)	C8B—C9B—C10B—C1B	−167.0 (6)
C8A—C9A—C10A—C5A	−50.7 (8)	C8B—C9B—C10B—C5B	−52.0 (8)
C8A—C9A—C10A—C25A	75.4 (8)	C8B—C9B—C10B—C25B	74.7 (8)
C8A—C9A—C11A—C12A	54.8 (8)	C8B—C9B—C11B—C12B	54.1 (9)
C8A—C14A—C15A—C16A	−162.0 (7)	C8B—C14B—C15B—C16B	−161.7 (6)
C9A—C8A—C14A—C13A	58.3 (7)	C9B—C8B—C14B—C13B	59.8 (7)
C9A—C8A—C14A—C15A	179.0 (6)	C9B—C8B—C14B—C15B	179.9 (6)
C9A—C8A—C14A—C27A	−61.1 (7)	C9B—C8B—C14B—C27B	−61.3 (7)
C9A—C11A—C12A—F1A	76.5 (7)	C9B—C11B—C12B—F1B	76.2 (8)
C9A—C11A—C12A—C13A	−41.2 (9)	C9B—C11B—C12B—C13B	−43.1 (10)
C10A—C1A—C2A—C3A	−57.9 (9)	C10B—C1B—C2B—C3B	−56.4 (8)
C10A—C5A—C6A—C7A	−62.0 (9)	C10B—C5B—C6B—C7B	−61.8 (8)
C10A—C9A—C11A—C12A	−169.7 (6)	C10B—C9B—C11B—C12B	−173.0 (7)
C11A—C9A—C10A—C1A	61.3 (8)	C11B—C9B—C10B—C1B	63.0 (8)
C11A—C9A—C10A—C5A	177.3 (6)	C11B—C9B—C10B—C5B	178.0 (6)
C11A—C9A—C10A—C25A	−56.6 (8)	C11B—C9B—C10B—C25B	−55.3 (8)
C11A—C12A—C13A—O1A	−79.9 (8)	C11B—C12B—C13B—O1B	−76.2 (8)
C11A—C12A—C13A—C14A	36.8 (9)	C11B—C12B—C13B—C14B	40.7 (10)
C11A—C12A—C13A—C18A	174.4 (6)	C11B—C12B—C13B—C18B	178.0 (7)
C12A—C13A—C14A—C8A	−46.2 (8)	C12B—C13B—C14B—C8B	−49.4 (8)
C12A—C13A—C14A—C15A	−168.5 (6)	C12B—C13B—C14B—C15B	−171.4 (6)
C12A—C13A—C14A—C27A	75.1 (8)	C12B—C13B—C14B—C27B	73.2 (8)
C12A—C13A—C18A—C17A	155.5 (6)	C12B—C13B—C18B—C17B	157.6 (6)
C12A—C13A—C18A—C19A	−77.5 (9)	C12B—C13B—C18B—C19B	−74.8 (9)
C13A—O1A—C28A—O2A	−176.9 (8)	C13B—O1B—C28B—O2B	−175.1 (8)
C13A—O1A—C28A—C17A	4.7 (8)	C13B—O1B—C28B—C17B	4.2 (9)
C13A—C14A—C15A—C16A	−39.8 (9)	C13B—C14B—C15B—C16B	−39.8 (8)
C13A—C18A—C19A—C20A	−179.4 (6)	C13B—C18B—C19B—C20B	−179.1 (7)
C14A—C8A—C9A—C10A	163.8 (6)	C14B—C8B—C9B—C10B	166.6 (6)
C14A—C8A—C9A—C11A	−62.1 (8)	C14B—C8B—C9B—C11B	−61.7 (7)
C14A—C13A—C18A—C17A	−67.5 (8)	C14B—C13B—C18B—C17B	−67.1 (8)
C14A—C13A—C18A—C19A	59.5 (9)	C14B—C13B—C18B—C19B	60.5 (10)
C14A—C15A—C16A—C17A	44.7 (9)	C14B—C15B—C16B—C17B	47.3 (9)
C15A—C16A—C17A—C18A	−60.8 (9)	C15B—C16B—C17B—C18B	−63.5 (8)
C15A—C16A—C17A—C22A	172.8 (6)	C15B—C16B—C17B—C22B	170.6 (6)
C15A—C16A—C17A—C28A	45.6 (8)	C15B—C16B—C17B—C28B	41.6 (9)
C16A—C17A—C18A—C13A	66.2 (8)	C16B—C17B—C18B—C13B	66.8 (7)
C16A—C17A—C18A—C19A	−70.3 (8)	C16B—C17B—C18B—C19B	−70.7 (8)
C16A—C17A—C22A—C21A	71.0 (8)	C16B—C17B—C22B—C21B	72.2 (9)
C16A—C17A—C28A—O1A	−88.5 (7)	C16B—C17B—C28B—O1B	−86.3 (8)
C16A—C17A—C28A—O2A	93.2 (11)	C16B—C17B—C28B—O2B	92.9 (11)
C17A—C18A—C19A—C20A	−57.3 (8)	C17B—C18B—C19B—C20B	−56.6 (8)
C18A—C13A—C14A—C8A	177.5 (6)	C18B—C13B—C14B—C8B	175.9 (6)
C18A—C13A—C14A—C15A	55.2 (8)	C18B—C13B—C14B—C15B	53.9 (8)
C18A—C13A—C14A—C27A	−61.2 (8)	C18B—C13B—C14B—C27B	−61.4 (8)
C18A—C17A—C22A—C21A	−55.1 (9)	C18B—C17B—C22B—C21B	−51.8 (9)
C18A—C17A—C28A—O1A	25.9 (8)	C18B—C17B—C28B—O1B	26.5 (8)
C18A—C17A—C28A—O2A	−152.4 (10)	C18B—C17B—C28B—O2B	−154.3 (10)
C18A—C19A—C20A—C21A	54.2 (9)	C18B—C19B—C20B—C21B	54.9 (8)
C18A—C19A—C20A—C29A	−68.7 (9)	C18B—C19B—C20B—C29B	−66.2 (8)
C18A—C19A—C20A—C30A	172.2 (7)	C18B—C19B—C20B—C30B	173.9 (7)
C19A—C20A—C21A—C22A	−54.1 (10)	C19B—C20B—C21B—C22B	−53.7 (9)
C20A—C21A—C22A—C17A	54.4 (9)	C20B—C21B—C22B—C17B	52.1 (9)
C22A—C17A—C18A—C13A	−165.5 (6)	C22B—C17B—C18B—C13B	−166.9 (6)
C22A—C17A—C18A—C19A	58.1 (9)	C22B—C17B—C18B—C19B	55.6 (8)
C22A—C17A—C28A—O1A	144.5 (6)	C22B—C17B—C28B—O1B	146.3 (7)
C22A—C17A—C28A—O2A	−33.8 (13)	C22B—C17B—C28B—O2B	−34.5 (13)
C23A—C4A—C5A—C6A	−60.2 (9)	C23B—C4B—C5B—C6B	−59.8 (8)
C23A—C4A—C5A—C10A	167.7 (7)	C23B—C4B—C5B—C10B	169.9 (6)
C24A—C4A—C5A—C6A	60.1 (9)	C24B—C4B—C5B—C6B	59.2 (8)
C24A—C4A—C5A—C10A	−72.0 (9)	C24B—C4B—C5B—C10B	−71.1 (8)
C26A—C8A—C9A—C10A	−72.9 (8)	C26B—C8B—C9B—C10B	−69.7 (8)
C26A—C8A—C9A—C11A	61.2 (8)	C26B—C8B—C9B—C11B	62.1 (8)
C26A—C8A—C14A—C13A	−63.2 (7)	C26B—C8B—C14B—C13B	−62.6 (8)
C26A—C8A—C14A—C15A	57.5 (8)	C26B—C8B—C14B—C15B	57.5 (8)
C26A—C8A—C14A—C27A	177.4 (6)	C26B—C8B—C14B—C27B	176.3 (6)
C27A—C14A—C15A—C16A	76.4 (8)	C27B—C14B—C15B—C16B	76.8 (8)
C28A—O1A—C13A—C12A	−149.1 (6)	C28B—O1B—C13B—C12B	−148.7 (7)
C28A—O1A—C13A—C14A	88.9 (7)	C28B—O1B—C13B—C14B	90.1 (7)
C28A—O1A—C13A—C18A	−32.4 (7)	C28B—O1B—C13B—C18B	−32.9 (7)
C28A—C17A—C18A—C13A	−44.2 (7)	C28B—C17B—C18B—C13B	−44.7 (7)
C28A—C17A—C18A—C19A	179.4 (6)	C28B—C17B—C18B—C19B	177.8 (6)
C28A—C17A—C22A—C21A	−166.9 (7)	C28B—C17B—C22B—C21B	−163.3 (7)
C29A—C20A—C21A—C22A	69.3 (9)	C29B—C20B—C21B—C22B	69.3 (8)
C30A—C20A—C21A—C22A	−171.8 (7)	C30B—C20B—C21B—C22B	−172.1 (7)
F1D—C12D—C13D—O1D	161.7 (5)	F1C—C12C—C13C—O1C	163.8 (6)
F1D—C12D—C13D—C14D	−81.3 (8)	F1C—C12C—C13C—C14C	−79.7 (8)
F1D—C12D—C13D—C18D	56.7 (8)	F1C—C12C—C13C—C18C	57.3 (8)
O1D—C13D—C14D—C8D	68.0 (7)	O1C—C13C—C14C—C8C	65.3 (7)
O1D—C13D—C14D—C15D	−53.6 (8)	O1C—C13C—C14C—C15C	−56.1 (8)
O1D—C13D—C14D—C27D	−170.5 (6)	O1C—C13C—C14C—C27C	−171.6 (6)
O1D—C13D—C18D—C17D	46.1 (6)	O1C—C13C—C18C—C17C	47.3 (6)
O1D—C13D—C18D—C19D	172.4 (6)	O1C—C13C—C18C—C19C	177.2 (7)
O3D—C3D—C4D—C5D	179.0 (6)	O3C—C3C—C4C—C5C	−175.9 (6)
O3D—C3D—C4D—C23D	62.1 (8)	O3C—C3C—C4C—C23C	66.0 (8)
O3D—C3D—C4D—C24D	−53.6 (9)	O3C—C3C—C4C—C24C	−51.1 (8)
C1D—C2D—C3D—O3D	−174.5 (6)	C1C—C2C—C3C—O3C	178.4 (6)
C1D—C2D—C3D—C4D	57.0 (9)	C1C—C2C—C3C—C4C	55.4 (9)
C2D—C1D—C10D—C5D	51.5 (8)	C2C—C1C—C10C—C5C	54.2 (8)
C2D—C1D—C10D—C9D	166.9 (6)	C2C—C1C—C10C—C9C	168.4 (6)
C2D—C1D—C10D—C25D	−72.6 (8)	C2C—C1C—C10C—C25C	−69.5 (7)
C2D—C3D—C4D—C5D	−52.6 (8)	C2C—C3C—C4C—C5C	−51.9 (8)
C2D—C3D—C4D—C23D	−169.6 (7)	C2C—C3C—C4C—C23C	−170.0 (7)
C2D—C3D—C4D—C24D	74.8 (9)	C2C—C3C—C4C—C24C	72.9 (8)
C3D—C4D—C5D—C6D	−176.3 (7)	C3C—C4C—C5C—C6C	−175.3 (6)
C3D—C4D—C5D—C10D	52.4 (8)	C3C—C4C—C5C—C10C	53.2 (8)
C4D—C5D—C6D—C7D	162.2 (6)	C4C—C5C—C6C—C7C	164.7 (6)
C4D—C5D—C10D—C1D	−52.0 (9)	C4C—C5C—C10C—C1C	−53.4 (8)
C4D—C5D—C10D—C9D	−168.0 (6)	C4C—C5C—C10C—C9C	−169.3 (6)
C4D—C5D—C10D—C25D	66.7 (9)	C4C—C5C—C10C—C25C	65.6 (8)
C5D—C6D—C7D—C8D	54.4 (9)	C5C—C6C—C7C—C8C	55.9 (8)
C6D—C5D—C10D—C1D	174.8 (6)	C6C—C5C—C10C—C1C	173.8 (6)
C6D—C5D—C10D—C9D	58.8 (8)	C6C—C5C—C10C—C9C	57.8 (7)
C6D—C5D—C10D—C25D	−66.5 (9)	C6C—C5C—C10C—C25C	−67.3 (8)
C6D—C7D—C8D—C9D	−42.7 (9)	C6C—C7C—C8C—C9C	−47.6 (8)
C6D—C7D—C8D—C14D	−161.2 (6)	C6C—C7C—C8C—C14C	−164.4 (6)
C6D—C7D—C8D—C26D	76.6 (8)	C6C—C7C—C8C—C26C	73.1 (8)
C7D—C8D—C9D—C10D	42.9 (9)	C7C—C8C—C9C—C10C	48.5 (8)
C7D—C8D—C9D—C11D	176.6 (6)	C7C—C8C—C9C—C11C	178.6 (6)
C7D—C8D—C14D—C13D	179.3 (6)	C7C—C8C—C14C—C13C	−179.9 (6)
C7D—C8D—C14D—C15D	−60.3 (8)	C7C—C8C—C14C—C15C	−59.9 (7)
C7D—C8D—C14D—C27D	58.6 (8)	C7C—C8C—C14C—C27C	58.7 (7)
C8D—C9D—C10D—C1D	−166.7 (6)	C8C—C9C—C10C—C1C	−167.4 (6)
C8D—C9D—C10D—C5D	−50.7 (8)	C8C—C9C—C10C—C5C	−53.0 (7)
C8D—C9D—C10D—C25D	76.3 (8)	C8C—C9C—C10C—C25C	73.6 (7)
C8D—C9D—C11D—C12D	55.9 (8)	C8C—C9C—C11C—C12C	55.8 (8)
C8D—C14D—C15D—C16D	−160.9 (7)	C8C—C14C—C15C—C16C	−162.0 (6)
C9D—C8D—C14D—C13D	58.1 (8)	C9C—C8C—C14C—C13C	61.2 (7)
C9D—C8D—C14D—C15D	178.5 (6)	C9C—C8C—C14C—C15C	−178.9 (5)
C9D—C8D—C14D—C27D	−62.6 (7)	C9C—C8C—C14C—C27C	−60.3 (7)
C9D—C11D—C12D—F1D	76.8 (7)	C9C—C11C—C12C—F1C	75.4 (8)
C9D—C11D—C12D—C13D	−42.0 (10)	C9C—C11C—C12C—C13C	−44.3 (9)
C10D—C1D—C2D—C3D	−56.5 (9)	C10C—C1C—C2C—C3C	−57.5 (8)
C10D—C5D—C6D—C7D	−63.2 (9)	C10C—C5C—C6C—C7C	−61.4 (8)
C10D—C9D—C11D—C12D	−168.7 (6)	C10C—C9C—C11C—C12C	−172.8 (6)
C11D—C9D—C10D—C1D	61.4 (8)	C11C—C9C—C10C—C1C	64.8 (7)
C11D—C9D—C10D—C5D	177.3 (6)	C11C—C9C—C10C—C5C	179.2 (6)
C11D—C9D—C10D—C25D	−55.6 (8)	C11C—C9C—C10C—C25C	−54.1 (8)
C11D—C12D—C13D—O1D	−78.7 (8)	C11C—C12C—C13C—O1C	−75.3 (7)
C11D—C12D—C13D—C14D	38.3 (10)	C11C—C12C—C13C—C14C	41.1 (9)
C11D—C12D—C13D—C18D	176.3 (7)	C11C—C12C—C13C—C18C	178.2 (6)
C12D—C13D—C14D—C8D	−46.2 (9)	C12C—C13C—C14C—C8C	−49.9 (8)
C12D—C13D—C14D—C15D	−167.8 (7)	C12C—C13C—C14C—C15C	−171.3 (6)
C12D—C13D—C14D—C27D	75.3 (8)	C12C—C13C—C14C—C27C	73.2 (8)
C12D—C13D—C18D—C17D	153.7 (6)	C12C—C13C—C18C—C17C	157.8 (6)
C12D—C13D—C18D—C19D	−79.9 (9)	C12C—C13C—C18C—C19C	−72.3 (9)
C13D—O1D—C28D—O2D	−174.1 (7)	C13C—O1C—C28C—O2C	−174.4 (8)
C13D—O1D—C28D—C17D	4.8 (8)	C13C—O1C—C28C—C17C	3.8 (8)
C13D—C14D—C15D—C16D	−39.4 (9)	C13C—C14C—C15C—C16C	−40.6 (9)
C13D—C18D—C19D—C20D	−179.0 (6)	C13C—C18C—C19C—C20C	−179.6 (7)
C14D—C8D—C9D—C10D	163.2 (6)	C14C—C8C—C9C—C10C	167.2 (5)
C14D—C8D—C9D—C11D	−63.1 (8)	C14C—C8C—C9C—C11C	−62.8 (7)
C14D—C13D—C18D—C17D	−68.2 (8)	C14C—C13C—C18C—C17C	−67.1 (7)
C14D—C13D—C18D—C19D	58.1 (9)	C14C—C13C—C18C—C19C	62.8 (10)
C14D—C15D—C16D—C17D	43.4 (10)	C14C—C15C—C16C—C17C	48.1 (9)
C15D—C16D—C17D—C18D	−59.1 (9)	C15C—C16C—C17C—C18C	−64.6 (8)
C15D—C16D—C17D—C22D	174.7 (6)	C15C—C16C—C17C—C22C	170.4 (6)
C15D—C16D—C17D—C28D	46.7 (8)	C15C—C16C—C17C—C28C	41.8 (9)
C16D—C17D—C18D—C13D	66.5 (7)	C16C—C17C—C18C—C13C	67.8 (7)
C16D—C17D—C18D—C19D	−68.9 (8)	C16C—C17C—C18C—C19C	−71.0 (8)
C16D—C17D—C22D—C21D	70.1 (8)	C16C—C17C—C22C—C21C	72.0 (9)
C16D—C17D—C28D—O1D	−88.9 (7)	C16C—C17C—C28C—O1C	−86.4 (8)
C16D—C17D—C28D—O2D	89.9 (10)	C16C—C17C—C28C—O2C	91.6 (11)
C17D—C18D—C19D—C20D	−57.4 (8)	C17C—C18C—C19C—C20C	−55.2 (8)
C18D—C13D—C14D—C8D	176.9 (6)	C18C—C13C—C14C—C8C	174.9 (6)
C18D—C13D—C14D—C15D	55.3 (8)	C18C—C13C—C14C—C15C	53.5 (8)
C18D—C13D—C14D—C27D	−61.6 (8)	C18C—C13C—C14C—C27C	−62.1 (8)
C18D—C17D—C22D—C21D	−55.7 (8)	C18C—C17C—C22C—C21C	−51.1 (9)
C18D—C17D—C28D—O1D	25.0 (7)	C18C—C17C—C28C—O1C	26.8 (8)
C18D—C17D—C28D—O2D	−156.2 (8)	C18C—C17C—C28C—O2C	−155.2 (9)
C18D—C19D—C20D—C21D	52.9 (8)	C18C—C19C—C20C—C21C	54.3 (8)
C18D—C19D—C20D—C29D	−71.7 (8)	C18C—C19C—C20C—C29C	−65.9 (8)
C18D—C19D—C20D—C30D	170.8 (7)	C18C—C19C—C20C—C30C	174.8 (6)
C19D—C20D—C21D—C22D	−52.3 (9)	C19C—C20C—C21C—C22C	−55.2 (8)
C20D—C21D—C22D—C17D	53.5 (9)	C20C—C21C—C22C—C17C	53.1 (9)
C22D—C17D—C18D—C13D	−165.0 (6)	C22C—C17C—C18C—C13C	−167.2 (6)
C22D—C17D—C18D—C19D	59.6 (8)	C22C—C17C—C18C—C19C	54.0 (8)
C22D—C17D—C28D—O1D	143.3 (6)	C22C—C17C—C28C—O1C	146.8 (7)
C22D—C17D—C28D—O2D	−37.9 (12)	C22C—C17C—C28C—O2C	−35.2 (13)
C23D—C4D—C5D—C6D	−61.0 (9)	C23C—C4C—C5C—C6C	−58.0 (8)
C23D—C4D—C5D—C10D	167.7 (7)	C23C—C4C—C5C—C10C	170.5 (6)
C24D—C4D—C5D—C6D	58.5 (9)	C24C—C4C—C5C—C6C	61.1 (8)
C24D—C4D—C5D—C10D	−72.8 (9)	C24C—C4C—C5C—C10C	−70.5 (8)
C26D—C8D—C9D—C10D	−74.0 (8)	C26C—C8C—C9C—C10C	−68.5 (7)
C26D—C8D—C9D—C11D	59.7 (8)	C26C—C8C—C9C—C11C	61.5 (8)
C26D—C8D—C14D—C13D	−62.9 (8)	C26C—C8C—C14C—C13C	−62.0 (8)
C26D—C8D—C14D—C15D	57.5 (8)	C26C—C8C—C14C—C15C	57.9 (8)
C26D—C8D—C14D—C27D	176.4 (6)	C26C—C8C—C14C—C27C	176.5 (6)
C27D—C14D—C15D—C16D	78.5 (8)	C27C—C14C—C15C—C16C	76.4 (8)
C28D—O1D—C13D—C12D	−148.4 (6)	C28C—O1C—C13C—C12C	−149.5 (7)
C28D—O1D—C13D—C14D	88.7 (7)	C28C—O1C—C13C—C14C	90.0 (7)
C28D—O1D—C13D—C18D	−32.1 (7)	C28C—O1C—C13C—C18C	−32.2 (7)
C28D—C17D—C18D—C13D	−43.3 (7)	C28C—C17C—C18C—C13C	−44.9 (6)
C28D—C17D—C18D—C19D	−178.7 (6)	C28C—C17C—C18C—C19C	176.3 (6)
C28D—C17D—C22D—C21D	−167.2 (7)	C28C—C17C—C22C—C21C	−163.4 (7)
C29D—C20D—C21D—C22D	72.3 (9)	C29C—C20C—C21C—C22C	66.8 (8)
C30D—C20D—C21D—C22D	−169.9 (7)	C30C—C20C—C21C—C22C	−175.4 (7)

### 12-α-Fluoro-3β-hydroxyolean-28,13β-olide methanol hemisolvate (1) Hydrogen-bond geometry (Å, º)

**Table:**

*D*—H···*A*	*D*—H	H···*A*	*D*···*A*	*D*—H···*A*
O13—H13···O3*A*	0.82	1.93	2.720 (8)	161
O14—H14···O3*D*^i^	0.82	1.94	2.714 (9)	157
O3*A*—H3*A*···O3*C*^ii^	0.82	2.03	2.819 (8)	163
O3*D*—H3*D*···O3*B*^iii^	0.82	2.01	2.816 (8)	166
O3*B*—H3*B*···O14^iv^	0.82	2.01	2.715 (8)	144
O3*C*—H3*C*···O13^ii^	0.82	2.07	2.708 (9)	135

Symmetry codes: (i) *x*+1, *y*−1, *z*; (ii) −*x*+1, *y*+1/2, −*z*+1; (iii) −*x*, *y*+1/2, −*z*; (iv) −*x*+1, *y*−1/2, −*z*.

### 12-α-Fluoro-3β-hydroxytaraxer-28,14β-olide methanol hemisolvate (2) Crystal data

**Table:**

2C_30_H_47_FO_3_·CH_4_O	*D*_x_ = 1.199 Mg m^−^^3^
*M**_r_* = 981.39	Cu *K*α radiation, λ = 1.54184 Å
Orthorhombic, *C*222_1_	Cell parameters from 8721 reflections
*a* = 6.6077 (2) Å	θ = 4.5–70.2°
*b* = 13.8730 (4) Å	µ = 0.64 mm^−^^1^
*c* = 59.326 (2) Å	*T* = 240 K
*V* = 5438.3 (3) Å^3^	Block, clear light colourless
*Z* = 4	0.34 × 0.2 × 0.1 mm
*F*(000) = 2152	

### 12-α-Fluoro-3β-hydroxytaraxer-28,14β-olide methanol hemisolvate (2) Data collection

**Table:**

XtaLAB AFC11 (RINC): Kappa single diffractometer	5184 independent reflections
Radiation source: Rotating-anode X-ray tube (dual wavelength), Rigaku (Cu) X-ray DW Source	4937 reflections with *I* > 2σ(*I*)
Mirror monochromator	*R*_int_ = 0.059
Detector resolution: 10.0000 pixels mm^-1^	θ_max_ = 70.8°, θ_min_ = 1.5°
ω scans	*h* = −7→8
Absorption correction: multi-scan (CrysAlisPro; Rigaku OD, 2020)	*k* = −16→16
*T*_min_ = 0.907, *T*_max_ = 1.000	*l* = −72→72
24920 measured reflections	

### 12-α-Fluoro-3β-hydroxytaraxer-28,14β-olide methanol hemisolvate (2) Refinement

**Table:**

Refinement on *F*^2^	Hydrogen site location: mixed
Least-squares matrix: full	H atoms treated by a mixture of independent and constrained refinement
*R*[*F*^2^ > 2σ(*F*^2^)] = 0.073	*w* = 1/[σ^2^(*F*_o_^2^) + (0.0838*P*)^2^ + 12.3803*P*] where *P* = (*F*_o_^2^ + 2*F*_c_^2^)/3
*wR*(*F*^2^) = 0.203	(Δ/σ)_max_ < 0.001
*S* = 1.09	Δρ_max_ = 0.27 e Å^−^^3^
5184 reflections	Δρ_min_ = −0.28 e Å^−^^3^
328 parameters	Absolute structure: Refined as an inversion twin
0 restraints	Absolute structure parameter: 0.5 (5)
Primary atom site location: structure-invariant direct methods	

### 12-α-Fluoro-3β-hydroxytaraxer-28,14β-olide methanol hemisolvate (2) Special details

**Table:**

Geometry. All esds (except the esd in the dihedral angle between two l.s. planes) are estimated using the full covariance matrix. The cell esds are taken into account individually in the estimation of esds in distances, angles and torsion angles; correlations between esds in cell parameters are only used when they are defined by crystal symmetry. An approximate (isotropic) treatment of cell esds is used for estimating esds involving l.s. planes.
Refinement. Refined as a 2-component inversion twin.

### 12-α-Fluoro-3β-hydroxytaraxer-28,14β-olide methanol hemisolvate (2) Fractional atomic coordinates and isotropic or equivalent isotropic displacement parameters (Å^2^)

**Table:**

	*x*	*y*	*z*	*U*_iso_*/*U*_eq_	Occ. (<1)
F1	0.2840 (7)	0.0871 (2)	0.60866 (5)	0.0576 (10)	
O1	0.0832 (6)	0.3645 (3)	0.59306 (6)	0.0436 (8)	
O2	−0.0826 (7)	0.3722 (4)	0.56100 (7)	0.0658 (13)	
O3	0.6529 (8)	0.3849 (3)	0.73386 (7)	0.0665 (13)	
H3A	0.555785	0.411829	0.739752	0.100*	0.5
H3B	0.773505	0.380509	0.737472	0.100*	0.5
C1	0.4758 (9)	0.2447 (4)	0.68283 (9)	0.0437 (12)	
H1A	0.412955	0.180899	0.681726	0.052*	
H1B	0.604846	0.242451	0.674715	0.052*	
C2	0.5162 (11)	0.2679 (4)	0.70767 (9)	0.0525 (14)	
H2A	0.388398	0.265705	0.716037	0.063*	
H2B	0.606485	0.218855	0.714021	0.063*	
C3	0.6117 (9)	0.3666 (4)	0.71045 (9)	0.0472 (13)	
H3	0.743471	0.365217	0.702509	0.057*	
C4	0.4848 (9)	0.4487 (4)	0.69983 (9)	0.0441 (12)	
C5	0.4351 (8)	0.4211 (3)	0.67510 (8)	0.0379 (11)	
H5	0.568105	0.416961	0.667475	0.045*	
C6	0.3173 (11)	0.4984 (4)	0.66195 (9)	0.0508 (14)	
H6A	0.176214	0.499208	0.667036	0.061*	
H6B	0.376097	0.561918	0.664967	0.061*	
C7	0.3242 (10)	0.4781 (4)	0.63663 (9)	0.0469 (13)	
H7A	0.464819	0.482864	0.631483	0.056*	
H7B	0.246082	0.527804	0.628767	0.056*	
C8	0.2402 (8)	0.3780 (3)	0.63000 (8)	0.0373 (11)	
C9	0.3416 (8)	0.3015 (3)	0.64534 (8)	0.0355 (10)	
H9	0.493 (10)	0.315 (4)	0.6416 (9)	0.043*	
C10	0.3357 (8)	0.3201 (3)	0.67148 (8)	0.0358 (10)	
C11	0.2845 (9)	0.1979 (4)	0.63895 (9)	0.0431 (12)	
H11A	0.394875	0.154761	0.643421	0.052*	
H11B	0.163779	0.179076	0.647491	0.052*	
C12	0.2426 (9)	0.1840 (4)	0.61396 (8)	0.0419 (12)	
H12	0.096772	0.195886	0.611298	0.050*	
C13	0.3628 (7)	0.2502 (4)	0.59854 (8)	0.0368 (10)	
C14	0.2859 (7)	0.3553 (4)	0.60376 (8)	0.0356 (10)	
C15	0.4196 (9)	0.4272 (3)	0.59122 (9)	0.0411 (11)	
H15A	0.555215	0.427729	0.597902	0.049*	
H15B	0.362401	0.492109	0.592596	0.049*	
C16	0.4338 (9)	0.3991 (4)	0.56613 (9)	0.0429 (12)	
H16A	0.568412	0.372634	0.562980	0.051*	
H16B	0.415098	0.456535	0.556756	0.051*	
C17	0.2702 (8)	0.3231 (4)	0.56014 (9)	0.0407 (11)	
C18	0.3136 (8)	0.2272 (3)	0.57298 (8)	0.0373 (10)	
H18	0.186524	0.189474	0.572817	0.045*	
C19	0.4753 (9)	0.1659 (4)	0.56080 (9)	0.0444 (12)	
H19A	0.607020	0.197458	0.562558	0.053*	
H19B	0.483375	0.102941	0.568224	0.053*	
C20	0.4361 (10)	0.1499 (4)	0.53556 (9)	0.0504 (14)	
C21	0.4139 (10)	0.2492 (4)	0.52433 (9)	0.0509 (13)	
H21A	0.542027	0.284295	0.525676	0.061*	
H21B	0.385569	0.240318	0.508249	0.061*	
C22	0.2463 (10)	0.3086 (5)	0.53479 (9)	0.0500 (13)	
H22A	0.116499	0.276830	0.531885	0.060*	
H22B	0.242806	0.371881	0.527445	0.060*	
C23	0.6245 (14)	0.5380 (5)	0.69954 (11)	0.071 (2)	
H23A	0.731204	0.528546	0.688528	0.106*	
H23B	0.683808	0.546927	0.714345	0.106*	
H23C	0.546124	0.594612	0.695547	0.106*	
C24	0.3007 (12)	0.4738 (5)	0.71449 (10)	0.0593 (17)	
H24A	0.209024	0.514591	0.706002	0.089*	
H24B	0.345452	0.507731	0.727897	0.089*	
H24C	0.231465	0.415052	0.718841	0.089*	
C25	0.1217 (8)	0.3094 (5)	0.68184 (9)	0.0474 (13)	
H25A	0.133305	0.288153	0.697362	0.071*	
H25B	0.045136	0.262255	0.673283	0.071*	
H25C	0.052551	0.371039	0.681352	0.071*	
C26	0.0101 (9)	0.3819 (5)	0.63399 (10)	0.0543 (15)	
H26A	−0.047827	0.318591	0.631411	0.081*	
H26B	−0.050452	0.427869	0.623678	0.081*	
H26C	−0.016472	0.401676	0.649389	0.081*	
C27	0.5939 (8)	0.2380 (4)	0.60303 (9)	0.0411 (11)	
H27A	0.669569	0.278113	0.592618	0.062*	
H27B	0.631646	0.171074	0.600896	0.062*	
H27C	0.624331	0.257290	0.618382	0.062*	
C28	0.0753 (8)	0.3567 (4)	0.57053 (9)	0.0431 (12)	
C29	0.2449 (13)	0.0879 (5)	0.53177 (11)	0.0668 (19)	
H29A	0.264519	0.024798	0.538476	0.100*	
H29B	0.220822	0.080823	0.515726	0.100*	
H29C	0.129351	0.119089	0.538726	0.100*	
C30	0.6189 (14)	0.0987 (6)	0.52549 (12)	0.077 (2)	
H30A	0.738613	0.138208	0.527568	0.115*	
H30B	0.596667	0.088149	0.509515	0.115*	
H30C	0.637947	0.037183	0.532961	0.115*	
O4	0.000000	0.3097 (5)	0.750000	0.091 (2)	
H4	0.117000	0.329434	0.749890	0.136*	0.5
C31	0.000000	0.2096 (7)	0.750000	0.071 (3)	
H31A	0.037080	0.186569	0.735310	0.085*	0.5
H31B	−0.132740	0.186569	0.753770	0.085*	0.5
H31C	0.095660	0.186569	0.760920	0.085*	0.5

### 12-α-Fluoro-3β-hydroxytaraxer-28,14β-olide methanol hemisolvate (2) Atomic displacement parameters (Å^2^)

**Table:**

	*U*^11^	*U*^22^	*U*^33^	*U*^12^	*U*^13^	*U*^23^
F1	0.087 (3)	0.0345 (15)	0.0507 (18)	−0.0143 (17)	−0.0015 (17)	0.0007 (13)
O1	0.0282 (17)	0.055 (2)	0.0472 (19)	0.0035 (16)	−0.0022 (15)	0.0099 (16)
O2	0.039 (2)	0.106 (4)	0.053 (2)	0.011 (2)	−0.0054 (19)	0.012 (2)
O3	0.083 (3)	0.071 (3)	0.046 (2)	0.005 (3)	−0.013 (2)	−0.009 (2)
C1	0.053 (3)	0.034 (2)	0.045 (3)	0.004 (2)	0.000 (2)	0.003 (2)
C2	0.065 (4)	0.050 (3)	0.043 (3)	0.015 (3)	−0.005 (3)	0.005 (2)
C3	0.041 (3)	0.061 (3)	0.040 (3)	0.002 (3)	0.000 (2)	−0.005 (2)
C4	0.049 (3)	0.043 (3)	0.041 (3)	−0.002 (2)	0.005 (2)	−0.002 (2)
C5	0.041 (3)	0.032 (2)	0.041 (3)	0.000 (2)	0.007 (2)	0.0018 (19)
C6	0.076 (4)	0.032 (2)	0.045 (3)	0.001 (3)	0.003 (3)	0.004 (2)
C7	0.068 (4)	0.028 (2)	0.045 (3)	0.004 (2)	0.000 (3)	0.005 (2)
C8	0.033 (3)	0.037 (2)	0.042 (3)	0.004 (2)	−0.002 (2)	0.006 (2)
C9	0.035 (3)	0.029 (2)	0.042 (2)	−0.0049 (19)	0.002 (2)	0.0055 (19)
C10	0.038 (3)	0.028 (2)	0.042 (2)	−0.0011 (19)	0.000 (2)	0.0054 (18)
C11	0.049 (3)	0.035 (2)	0.046 (3)	−0.004 (2)	0.002 (2)	0.005 (2)
C12	0.048 (3)	0.035 (2)	0.043 (3)	−0.012 (2)	0.000 (2)	0.005 (2)
C13	0.034 (3)	0.036 (2)	0.040 (3)	−0.003 (2)	−0.0015 (19)	0.0046 (19)
C14	0.029 (2)	0.037 (2)	0.041 (2)	−0.002 (2)	−0.0042 (19)	0.006 (2)
C15	0.040 (3)	0.033 (2)	0.050 (3)	−0.005 (2)	0.001 (2)	0.008 (2)
C16	0.043 (3)	0.043 (3)	0.043 (3)	−0.005 (2)	0.003 (2)	0.012 (2)
C17	0.035 (3)	0.044 (3)	0.044 (3)	0.002 (2)	−0.002 (2)	0.007 (2)
C18	0.033 (2)	0.038 (2)	0.041 (3)	0.002 (2)	0.000 (2)	0.005 (2)
C19	0.046 (3)	0.044 (3)	0.043 (3)	0.011 (2)	−0.002 (2)	0.006 (2)
C20	0.062 (4)	0.048 (3)	0.042 (3)	0.009 (3)	0.000 (3)	0.003 (2)
C21	0.062 (4)	0.052 (3)	0.039 (3)	0.002 (3)	0.004 (3)	0.004 (2)
C22	0.048 (3)	0.057 (3)	0.046 (3)	0.009 (3)	−0.001 (3)	0.007 (2)
C23	0.099 (6)	0.058 (4)	0.056 (4)	−0.027 (4)	−0.001 (4)	−0.009 (3)
C24	0.081 (5)	0.053 (3)	0.044 (3)	0.019 (3)	0.008 (3)	−0.004 (3)
C25	0.037 (3)	0.060 (3)	0.045 (3)	−0.005 (3)	0.005 (2)	0.006 (2)
C26	0.043 (3)	0.074 (4)	0.045 (3)	0.013 (3)	0.005 (2)	0.005 (3)
C27	0.031 (2)	0.050 (3)	0.043 (3)	0.003 (2)	−0.004 (2)	0.004 (2)
C28	0.032 (3)	0.053 (3)	0.045 (3)	0.007 (2)	−0.006 (2)	0.009 (2)
C29	0.082 (5)	0.064 (4)	0.054 (4)	−0.013 (4)	−0.007 (3)	−0.003 (3)
C30	0.110 (7)	0.069 (4)	0.051 (4)	0.035 (4)	0.013 (4)	0.003 (3)
O4	0.110 (7)	0.057 (4)	0.106 (6)	0.000	−0.019 (5)	0.000
C31	0.069 (6)	0.058 (5)	0.084 (7)	0.000	−0.001 (5)	0.000

### 12-α-Fluoro-3β-hydroxytaraxer-28,14β-olide methanol hemisolvate (2) Geometric parameters (Å, º)

**Table:**

F1—C12	1.408 (6)	C16—H16A	0.9800
O1—C14	1.487 (6)	C16—H16B	0.9800
O1—C28	1.342 (6)	C16—C17	1.551 (8)
O2—C28	1.206 (7)	C17—C18	1.559 (7)
O3—H3A	0.8206	C17—C22	1.526 (7)
O3—H3B	0.8272	C17—C28	1.502 (7)
O3—C3	1.438 (6)	C18—H18	0.9900
C1—H1A	0.9800	C18—C19	1.545 (7)
C1—H1B	0.9800	C19—H19A	0.9800
C1—C2	1.531 (7)	C19—H19B	0.9800
C1—C10	1.550 (7)	C19—C20	1.535 (7)
C2—H2A	0.9800	C20—C21	1.537 (8)
C2—H2B	0.9800	C20—C29	1.545 (10)
C2—C3	1.517 (8)	C20—C30	1.523 (10)
C3—H3	0.9900	C21—H21A	0.9800
C3—C4	1.548 (8)	C21—H21B	0.9800
C4—C5	1.552 (7)	C21—C22	1.514 (8)
C4—C23	1.545 (9)	C22—H22A	0.9800
C4—C24	1.536 (9)	C22—H22B	0.9800
C5—H5	0.9900	C23—H23A	0.9700
C5—C6	1.537 (7)	C23—H23B	0.9700
C5—C10	1.562 (6)	C23—H23C	0.9700
C6—H6A	0.9800	C24—H24A	0.9700
C6—H6B	0.9800	C24—H24B	0.9700
C6—C7	1.529 (8)	C24—H24C	0.9700
C7—H7A	0.9800	C25—H25A	0.9700
C7—H7B	0.9800	C25—H25B	0.9700
C7—C8	1.546 (7)	C25—H25C	0.9700
C8—C9	1.550 (6)	C26—H26A	0.9700
C8—C14	1.616 (7)	C26—H26B	0.9700
C8—C26	1.540 (8)	C26—H26C	0.9700
C9—H9	1.04 (6)	C27—H27A	0.9700
C9—C10	1.573 (7)	C27—H27B	0.9700
C9—C11	1.533 (7)	C27—H27C	0.9700
C10—C25	1.549 (7)	C29—H29A	0.9700
C11—H11A	0.9800	C29—H29B	0.9700
C11—H11B	0.9800	C29—H29C	0.9700
C11—C12	1.520 (7)	C30—H30A	0.9700
C12—H12	0.9900	C30—H30B	0.9700
C12—C13	1.521 (7)	C30—H30C	0.9700
C13—C14	1.574 (7)	O4—H4^i^	0.8199
C13—C18	1.583 (7)	O4—H4	0.8200
C13—C27	1.560 (7)	O4—C31	1.389 (12)
C14—C15	1.527 (7)	C31—H31A	0.9601
C15—H15A	0.9800	C31—H31B	0.9600
C15—H15B	0.9800	C31—H31C	0.9600
C15—C16	1.541 (7)		
			
C28—O1—C14	117.0 (4)	C15—C16—C17	110.5 (4)
C3—O3—H3A	110.1	H16A—C16—H16B	108.1
C3—O3—H3B	114.8	C17—C16—H16A	109.5
H1A—C1—H1B	107.9	C17—C16—H16B	109.5
C2—C1—H1A	109.1	C16—C17—C18	109.8 (4)
C2—C1—H1B	109.1	C22—C17—C16	112.8 (4)
C2—C1—C10	112.4 (4)	C22—C17—C18	112.9 (4)
C10—C1—H1A	109.1	C28—C17—C16	107.0 (4)
C10—C1—H1B	109.1	C28—C17—C18	102.9 (4)
C1—C2—H2A	109.3	C28—C17—C22	110.9 (4)
C1—C2—H2B	109.3	C13—C18—H18	106.9
H2A—C2—H2B	108.0	C17—C18—C13	109.5 (4)
C3—C2—C1	111.5 (5)	C17—C18—H18	106.9
C3—C2—H2A	109.3	C19—C18—C13	114.7 (4)
C3—C2—H2B	109.3	C19—C18—C17	111.6 (4)
O3—C3—C2	110.1 (5)	C19—C18—H18	106.9
O3—C3—H3	107.3	C18—C19—H19A	108.6
O3—C3—C4	111.4 (4)	C18—C19—H19B	108.6
C2—C3—H3	107.3	H19A—C19—H19B	107.5
C2—C3—C4	113.2 (5)	C20—C19—C18	114.8 (4)
C4—C3—H3	107.3	C20—C19—H19A	108.6
C3—C4—C5	108.6 (4)	C20—C19—H19B	108.6
C23—C4—C3	105.7 (5)	C19—C20—C21	108.0 (5)
C23—C4—C5	108.3 (4)	C19—C20—C29	111.1 (5)
C24—C4—C3	111.5 (5)	C21—C20—C29	111.0 (5)
C24—C4—C5	115.1 (5)	C30—C20—C19	108.5 (5)
C24—C4—C23	107.3 (5)	C30—C20—C21	108.9 (5)
C4—C5—H5	105.0	C30—C20—C29	109.4 (6)
C4—C5—C10	116.1 (4)	C20—C21—H21A	109.1
C6—C5—C4	114.6 (4)	C20—C21—H21B	109.1
C6—C5—H5	105.0	H21A—C21—H21B	107.9
C6—C5—C10	110.0 (4)	C22—C21—C20	112.4 (5)
C10—C5—H5	105.0	C22—C21—H21A	109.1
C5—C6—H6A	109.5	C22—C21—H21B	109.1
C5—C6—H6B	109.5	C17—C22—H22A	108.8
H6A—C6—H6B	108.1	C17—C22—H22B	108.8
C7—C6—C5	110.8 (4)	C21—C22—C17	113.6 (5)
C7—C6—H6A	109.5	C21—C22—H22A	108.8
C7—C6—H6B	109.5	C21—C22—H22B	108.8
C6—C7—H7A	108.8	H22A—C22—H22B	107.7
C6—C7—H7B	108.8	C4—C23—H23A	109.5
C6—C7—C8	113.9 (4)	C4—C23—H23B	109.5
H7A—C7—H7B	107.7	C4—C23—H23C	109.5
C8—C7—H7A	108.8	H23A—C23—H23B	109.5
C8—C7—H7B	108.8	H23A—C23—H23C	109.5
C7—C8—C9	108.1 (4)	H23B—C23—H23C	109.5
C7—C8—C14	110.7 (4)	C4—C24—H24A	109.5
C9—C8—C14	110.6 (4)	C4—C24—H24B	109.5
C26—C8—C7	106.5 (5)	C4—C24—H24C	109.5
C26—C8—C9	111.1 (4)	H24A—C24—H24B	109.5
C26—C8—C14	109.8 (4)	H24A—C24—H24C	109.5
C8—C9—H9	100 (3)	H24B—C24—H24C	109.5
C8—C9—C10	117.1 (4)	C10—C25—H25A	109.5
C10—C9—H9	102 (3)	C10—C25—H25B	109.5
C11—C9—C8	113.0 (4)	C10—C25—H25C	109.5
C11—C9—H9	110 (3)	H25A—C25—H25B	109.5
C11—C9—C10	113.1 (4)	H25A—C25—H25C	109.5
C1—C10—C5	107.1 (4)	H25B—C25—H25C	109.5
C1—C10—C9	107.6 (4)	C8—C26—H26A	109.5
C5—C10—C9	105.8 (4)	C8—C26—H26B	109.5
C25—C10—C1	107.9 (4)	C8—C26—H26C	109.5
C25—C10—C5	114.6 (4)	H26A—C26—H26B	109.5
C25—C10—C9	113.4 (4)	H26A—C26—H26C	109.5
C9—C11—H11A	108.8	H26B—C26—H26C	109.5
C9—C11—H11B	108.8	C13—C27—H27A	109.5
H11A—C11—H11B	107.7	C13—C27—H27B	109.5
C12—C11—C9	113.8 (4)	C13—C27—H27C	109.5
C12—C11—H11A	108.8	H27A—C27—H27B	109.5
C12—C11—H11B	108.8	H27A—C27—H27C	109.5
F1—C12—C11	107.7 (4)	H27B—C27—H27C	109.5
F1—C12—H12	108.2	O1—C28—C17	113.6 (4)
F1—C12—C13	109.9 (4)	O2—C28—O1	119.1 (5)
C11—C12—H12	108.2	O2—C28—C17	127.2 (5)
C13—C12—C11	114.5 (4)	C20—C29—H29A	109.5
C13—C12—H12	108.2	C20—C29—H29B	109.5
C12—C13—C14	105.8 (4)	C20—C29—H29C	109.5
C12—C13—C18	110.3 (4)	H29A—C29—H29B	109.5
C12—C13—C27	110.1 (4)	H29A—C29—H29C	109.5
C14—C13—C18	108.0 (4)	H29B—C29—H29C	109.5
C27—C13—C14	112.5 (4)	C20—C30—H30A	109.5
C27—C13—C18	110.0 (4)	C20—C30—H30B	109.5
O1—C14—C8	103.1 (4)	C20—C30—H30C	109.5
O1—C14—C13	106.6 (4)	H30A—C30—H30B	109.5
O1—C14—C15	104.9 (4)	H30A—C30—H30C	109.5
C13—C14—C8	115.5 (4)	H30B—C30—H30C	109.5
C15—C14—C8	116.8 (4)	H4—O4—H4^i^	141.1
C15—C14—C13	108.8 (4)	C31—O4—H4^i^	109.459 (11)
C14—C15—H15A	109.7	C31—O4—H4	109.5
C14—C15—H15B	109.7	O4—C31—H31A	109.5
C14—C15—C16	109.9 (4)	O4—C31—H31B	109.5
H15A—C15—H15B	108.2	O4—C31—H31C	109.5
C16—C15—H15A	109.7	H31A—C31—H31B	109.5
C16—C15—H15B	109.7	H31A—C31—H31C	109.5
C15—C16—H16A	109.5	H31B—C31—H31C	109.5
C15—C16—H16B	109.5		
			
F1—C12—C13—C14	173.2 (4)	C12—C13—C18—C19	−101.2 (5)
F1—C12—C13—C18	56.6 (5)	C13—C14—C15—C16	51.5 (5)
F1—C12—C13—C27	−65.0 (6)	C13—C18—C19—C20	−175.6 (5)
O1—C14—C15—C16	−62.3 (5)	C14—O1—C28—O2	−173.5 (5)
O3—C3—C4—C5	−176.6 (5)	C14—O1—C28—C17	9.8 (6)
O3—C3—C4—C23	67.5 (6)	C14—C8—C9—C10	172.4 (4)
O3—C3—C4—C24	−48.8 (7)	C14—C8—C9—C11	−53.6 (5)
C1—C2—C3—O3	−178.4 (5)	C14—C13—C18—C17	17.3 (5)
C1—C2—C3—C4	56.1 (6)	C14—C13—C18—C19	143.7 (4)
C2—C1—C10—C5	55.1 (6)	C14—C15—C16—C17	13.8 (6)
C2—C1—C10—C9	168.5 (5)	C15—C16—C17—C18	−65.6 (5)
C2—C1—C10—C25	−68.7 (6)	C15—C16—C17—C22	167.5 (4)
C2—C3—C4—C5	−51.8 (6)	C15—C16—C17—C28	45.3 (5)
C2—C3—C4—C23	−167.7 (5)	C16—C17—C18—C13	46.3 (6)
C2—C3—C4—C24	76.0 (6)	C16—C17—C18—C19	−81.8 (5)
C3—C4—C5—C6	−177.3 (5)	C16—C17—C22—C21	76.2 (6)
C3—C4—C5—C10	52.7 (6)	C16—C17—C28—O1	−60.8 (6)
C4—C5—C6—C7	164.8 (5)	C16—C17—C28—O2	122.8 (6)
C4—C5—C10—C1	−54.3 (6)	C17—C18—C19—C20	−50.4 (6)
C4—C5—C10—C9	−168.9 (4)	C18—C13—C14—O1	43.2 (5)
C4—C5—C10—C25	65.3 (6)	C18—C13—C14—C8	157.0 (4)
C5—C6—C7—C8	57.5 (7)	C18—C13—C14—C15	−69.5 (5)
C6—C5—C10—C1	173.5 (4)	C18—C17—C22—C21	−48.9 (7)
C6—C5—C10—C9	58.9 (5)	C18—C17—C28—O1	54.9 (6)
C6—C5—C10—C25	−66.8 (5)	C18—C17—C28—O2	−121.5 (7)
C6—C7—C8—C9	−49.6 (6)	C18—C19—C20—C21	55.7 (7)
C6—C7—C8—C14	−170.8 (5)	C18—C19—C20—C29	−66.2 (6)
C6—C7—C8—C26	69.9 (6)	C18—C19—C20—C30	173.5 (5)
C7—C8—C9—C10	51.1 (6)	C19—C20—C21—C22	−57.5 (7)
C7—C8—C9—C11	−174.9 (5)	C20—C21—C22—C17	56.2 (7)
C7—C8—C14—O1	−107.5 (4)	C22—C17—C18—C13	173.1 (4)
C7—C8—C14—C13	136.6 (4)	C22—C17—C18—C19	45.0 (6)
C7—C8—C14—C15	6.8 (6)	C22—C17—C28—O1	175.8 (5)
C8—C9—C10—C1	−170.5 (4)	C22—C17—C28—O2	−0.6 (9)
C8—C9—C10—C5	−56.2 (5)	C23—C4—C5—C6	−63.0 (7)
C8—C9—C10—C25	70.2 (5)	C23—C4—C5—C10	167.0 (5)
C8—C9—C11—C12	30.9 (7)	C24—C4—C5—C6	57.0 (6)
C8—C14—C15—C16	−175.6 (4)	C24—C4—C5—C10	−73.0 (6)
C9—C8—C14—O1	132.7 (4)	C26—C8—C9—C10	−65.4 (6)
C9—C8—C14—C13	16.9 (6)	C26—C8—C9—C11	68.6 (6)
C9—C8—C14—C15	−112.9 (5)	C26—C8—C14—O1	9.7 (5)
C9—C11—C12—F1	153.5 (4)	C26—C8—C14—C13	−106.1 (5)
C9—C11—C12—C13	30.9 (7)	C26—C8—C14—C15	124.1 (5)
C10—C1—C2—C3	−58.2 (7)	C27—C13—C14—O1	164.8 (4)
C10—C5—C6—C7	−62.2 (6)	C27—C13—C14—C8	−81.3 (5)
C10—C9—C11—C12	166.9 (4)	C27—C13—C14—C15	52.2 (5)
C11—C9—C10—C1	55.5 (6)	C27—C13—C18—C17	−105.9 (5)
C11—C9—C10—C5	169.8 (4)	C27—C13—C18—C19	20.5 (6)
C11—C9—C10—C25	−63.8 (6)	C28—O1—C14—C8	174.9 (4)
C11—C12—C13—C14	−65.5 (5)	C28—O1—C14—C13	−63.0 (5)
C11—C12—C13—C18	178.0 (4)	C28—O1—C14—C15	52.3 (5)
C11—C12—C13—C27	56.3 (6)	C28—C17—C18—C13	−67.4 (5)
C12—C13—C14—O1	−74.9 (4)	C28—C17—C18—C19	164.6 (4)
C12—C13—C14—C8	38.9 (5)	C28—C17—C22—C21	−163.7 (5)
C12—C13—C14—C15	172.4 (4)	C29—C20—C21—C22	64.5 (6)
C12—C13—C18—C17	132.5 (5)	C30—C20—C21—C22	−175.0 (6)

Symmetry code: (i) −*x*, *y*, −*z*+3/2.

### 12-α-Fluoro-3β-hydroxytaraxer-28,14β-olide methanol hemisolvate (2) Hydrogen-bond geometry (Å, º)

**Table:**

*D*—H···*A*	*D*—H	H···*A*	*D*···*A*	*D*—H···*A*
O3—H3*A*···O3^ii^	0.82	2.12	2.784 (10)	138
O3—H3*B*···O4^iii^	0.83	1.94	2.695 (6)	152
O4—H4···O3^ii^	0.82	1.96	2.695 (6)	149

Symmetry codes: (ii) −*x*+1, *y*, −*z*+3/2; (iii) *x*+1, *y*, *z*.
